# Disability-adjusted life years associated with chronic comorbidities among people living with and without HIV: Estimating health burden in British Columbia, Canada

**DOI:** 10.1371/journal.pgph.0001138

**Published:** 2022-10-14

**Authors:** Ni Gusti Ayu Nanditha, Jielin Zhu, Lu Wang, Jacek Kopec, Robert S. Hogg, Julio S. G. Montaner, Viviane D. Lima

**Affiliations:** 1 British Columbia Centre for Excellence in HIV/AIDS, Vancouver, Canada; 2 Department of Medicine, Faculty of Medicine, University of British Columbia, Vancouver, Canada; 3 Arthritis Research Canada, Richmond, British Columbia, Canada; 4 School of Population and Public Health, University of British Columbia, Vancouver, British Columbia, Canada; 5 Faculty of Health Sciences, Simon Fraser University, Burnaby, British Columbia, Canada; University of Alabama at Birmingham, UNITED STATES

## Abstract

Life span of people living with HIV (PLWH) has increased dramatically with the advent of modern antiretroviral therapy. As a result, comorbidities have emerged as a significant concern in this population. To describe the burden of chronic comorbidities among PLWH and HIV-negative individuals in British Columbia (BC), Canada, we estimated disability-adjusted life years (DALYs) related to these comorbidities. Based on a population-based cohort in BC, antiretroviral-treated adult PLWH and 1:4 age-sex-matched HIV-negative controls were followed for ≥1 year during 2001–2012. DALYs combined years of life lost to premature mortality (YLLs) and due to disability (YLDs), and were estimated following the Global Burden of Diseases’ approaches. DALYs associated with non-AIDS-defining cancers, diabetes, osteoarthritis, hypertension, dementia, cardiovascular (CVD), kidney, liver and chronic obstructive pulmonary diseases were each measured for 2008–2012. Among PLWH, DALYs attributed to non-AIDS-related cancers were also estimated for 2013–2020. We observed that at baseline, our matched cohort consisted of 82% males with a median age of 40 years (25th-75th percentiles: 34–47). During 2008–2012, 7042 PLWH and 30,640 HIV-negative individuals were alive, where PLWH experienced a twofold higher DALYs associated with chronic comorbidities (770.2 years/1000 people [95% credible intervals: 710.2, 831.6] vs. 359.0 [336.0, 382.2]). Non-AIDS-defining cancers and CVD contributed the highest DALYs in both populations, driven by YLLs rather than YLDs. Among PLWH, we estimated increasing DALYs attributable to non-AIDS-defining cancers with 91.7 years/1000 people (77.4, 106.0) in 2013 vs. 97.6 (81.0, 115.2) in 2020. In this study, we showed that PLWH experience a disproportionate burden of chronic comorbidities compared to HIV-negative individuals. The observed disparities may relate to differential health behaviors, residual HIV-related inflammation, and ART-related toxicities. As aging shapes future healthcare needs, our findings highlight the need to enhance prevention and management of comorbidities as part of HIV care.

## Introduction

As advances in antiretroviral therapy (ART) have dramatically increased longevity among people living with HIV (PLWH), the increased burden of non-AIDS chronic comorbidities has emerged as a major concern [1–4]. Compared to the general population, PLWH had reportedly a higher risk of developing chronic comorbidities such as non-AIDS-related cancers, kidney, lung, and cardiovascular diseases [5, 6], which often co-occurred [7, 8]. In British Columbia (BC), Canada, where ART has been available free of charge since 1992 and more than 60% of known PLWH in 2020 were 50 years or older [9], chronic comorbidities were diagnosed up to 10 years earlier among PLWH than HIV-negative controls [10]. Chronic comorbidities have also increasingly become the predominant cause of death among PLWH [11, 12], with chronic comorbidities making up to 78% of all deaths among BC’s PLWH in 2017, an increase from 35% in 2004 [13].

Disability-adjusted life years (DALYs) are a comprehensive metric of disease burden, accounting for both morbidity and mortality [14]. DALYs quantify the gap between the perfect health and observed health status based on years of life lost to premature deaths (YLLs) and years of healthy life lost due to disability (YLDs). DALYs were constructed as part of the first annual Global Burden of Diseases (GBD) study in the early 1990s [15]. Under the collaboration between the Institute of Health Metrics and Evaluation and the World Health Organization, the GBD has since undergone annual updates and several methodological changes, with the latest iteration investigating the health effects of 369 diseases and injuries in 204 countries in 2019 [16, 17]. Countries have also adopted DALYs to estimate their nation’s overall and cause-specific disease burden [18–24], often projecting the future burden of diseases to aid policy making and clinical service planning [25–27]. Note that despite many settings adopting DALYs as a metric, methodological choices frequently differed to address disparities in data availability and quality across countries and diseases [28].

In this study, we estimated and compared the burden of nine chronic comorbidities among PLWH and a population of age-sex-matched HIV-negative controls in BC by estimating YLLs, YLDs and DALYs associated with these comorbidities during 2008–2012. Among PLWH, the study also estimated the disease burden associated with non-AIDS-defining cancers from 2013 to 2020. To achieve these aims, we used a population-based cohort with linkages to key provincial health databases. We supplemented the GBD methodology with robust methodological approaches that catered to the BC setting to address potential biases arising from comparing PLWH and HIV-negative controls, and the use of administrative data for health research.

## Methods

### Ethics statement

Ethics approval for the COAST study was granted from the University of British Columbia/Providence Health Care Research Ethics Board (H09-02905; H16-02036) and Simon Fraser University Office of Research Ethics (#2013 s0566). The study complies with the BC Freedom of Information and Protection of Privacy Act (FIPPA) and did not require informed consent as it is conducted retrospectively for research and statistical purposes only using anonymized data.

### Data sources

We obtained longitudinal de-identified individual-level data from the Comparative Outcomes And Service Utilization Trends (COAST) study, which comprises all diagnosed adult PLWH and a 10% random sample of the general population in BC followed from 1996 to 2013 [29]. COAST was established through confidential linkages between two data sources: i) the BC Centre for Excellence in HIV/AIDS Drug Treatment Program (DTP), which provides demographic, treatment and laboratory information of all ART-treated PLWH in BC [30]; and ii) Population Data BC, which houses various provincial administrative health data of all BC residents [31–36]. COAST and associated data linkages have been detailed elsewhere [29]. While the abovementioned linkages between the DTP and provincial administrative datasets, as part of COAST, ended on March 31, 2013, the DTP dataset has been updated beyond this period. We thus obtained demographic data (i.e., age and sex at birth) of PLWH initiating and receiving ART through the DTP in BC during 2013–2020 from the DTP dataset.

### Study design

In this population-based matched-cohort study, eligible participants were aged ≥19 years and followed for ≥1 year in COAST between January 1, 2001 and December 31, 2012. Each ART-treated PLWH was matched to four HIV-negative individuals by birth year and sex at birth (Note B in S1 Text). For PLWH, baseline was the latest among known positive HIV serostatus, 19^th^ birthday, five years since earliest administrative record (either provincial Medical Service Plan registration or the first identified healthcare encounter), or January 1, 2001. For HIV-negative controls, baseline date was assigned to match their paired PLWH. Participants were observed until death, loss-to-follow-up, or December 31, 2012, whichever was earliest.

For the period after 2012, we considered PLWH aged ≥19 years who received treatment through the DTP between January 1, 2013 and December 31, 2020. For these PLWH, baseline was the latest between the first treatment date and January 1, 2013. They were then followed for ≥1 year until the earliest among death, last contact date and December 31, 2020.

### Outcome variables

Our outcomes were YLLs, YLDs and DALYs associated with nine chronic comorbidities highly prevalent among PLWH and BC’s general population [10, 37]: cardiovascular diseases (CVD), kidney disease, liver disease, chronic obstructive pulmonary disease (COPD), non-AIDS-defining cancers (excluding Kaposi sarcoma, non-Hodgkin’s lymphoma and cervical cancers; hereinafter referred to as cancers), diabetes, osteoarthritis, hypertension, and Alzheimer’s and/or non-HIV-related dementia (Alzheimer’s/dementia). Following age-restrictions in the case-finding algorithms, individuals considered for hypertension, COPD and Alzheimer’s/dementia analyses were older than 20, 35 and 40 years, respectively. Whenever possible, we estimated YLLs, YLDs and DALYs following the methodology used in the GBD 2019 [16, 17].

#### YLDs

YLDs quantified time lived in states of suboptimal health as the sum of the number of prevalent cases at a time period multiplied by the corresponding disability weight [16, 17]. The BC Cancer Agency Registry identified cancer cases [35]. The remaining comorbidity diagnoses were ascertained by case-finding algorithms using the International Classification of Disease Ninth and Tenth Revisions (ICD 9/10) codes and Canada-wide Drug Identification Numbers (Table A in S1 Text), which have been utilized in previous publications [10, 38]. These algorithms were applied to provincial administrative datasets including hospitalizations and day-surgeries [32], physician visits, laboratory and diagnostic procedures [33], and prescription drug dispensation [36]. For each year, we measured period prevalence; a comorbidity was prevalent if an individual was alive for ≥1 day in a given year and an existing diagnosis was identified within a 5-year observation window (i.e., at any point that year and/or within four years prior) [10]. Each comorbidity was associated with severity-specific disability weights between 0 and 1, representing a numerical assessment of non-fatal health loss, where 0 equaled full health and 1 equaled death [16, 17]. We used disability weights from the GBD 2019 (Table B in S1 Text), derived from largescale surveys of the general population, as opposed to health experts, across different cultural contexts [39, 40]. Given administrative data’s inability to ascertain disease severity, to approximate comorbidity-specific severity distributions in our study populations, we conducted a literature review to obtain distributions from BC or comparable settings (Table B in S1 Text).

#### YLLs

YLLs measured time lost to premature death as the number of deaths during a time period multiplied by a standard life expectancy at the age of death, which represents the maximum life span of healthy individuals receiving appropriate health services [16, 17]. We used the theoretical minimum risk life table from the GBD 2019 (Table C in S1 Text), constructed based on the lowest observed age-specific mortality rates for both sexes from locations with populations over five million in 2016 [41]. The underlying cause of death, defined as the disease or injury that initiated the series of events leading directly to death, was collected through BC Vital Statistic Agency’s mortality database. For consistency between YLLs and YLDs, we captured comorbidity-specific deaths using the same ICD 10 codes used to ascertain prevalent cases for YLD calculations (Table A in S1 Text).

#### DALYs

DALYs associated with each comorbidity were calculated as the sum of corresponding YLLs and YLDs, where one DALY depicted one lost year of healthy life due to a specific cause [16, 17].

### Analytical approaches

Categorical variables were compared using the Fisher’s exact test and continuous variables were compared using the Kruskal-Wallis test [42]. Statistical analyses were performed using R software version 3.2.2 (R Core Team, Vienna, Austria) or SAS software version 9.4 (SAS, Cary, North Carolina, United States), while data simulations were coded in Python and conducted using the NUMPY library [43]. This study consisted of a two-pronged analysis. Of note, our data were exhaustive (i.e., no missing values), with all eligible individuals included in each analysis.

#### Comparison of burden of chronic comorbidities among PLWH and HIV-negative controls, 2008–2012

We compared YLLs, YLDs and DALYs, overall and per 1000 people, associated with the abovementioned chronic comorbidities across HIV status for a combined 2008–2012 period. The combined estimates were chosen to ensure sufficient observations, particularly of mortality cases. Further, to ensure combined estimates that are most contemporary, we restricted our analyses to the last five years of COAST data (i.e., 2008–2012). To measure uncertainties around the point estimate of each outcome, the Monte Carlo simulation approach was used to estimate the 95% credible interval (CrI) [44]. Unlike a confidence interval which derived from hypothesized repeats of an experiment, a CrI illustrates a range containing a percentage (e.g., 95%) of probable estimates based on observed data [45]. Hence, the lower and upper limits of a 95% CrI are the 2.5th and 97.5th percentiles, within which there is a 95% probability that the true estimate may lie given the evidence provided by our observed data. For YLLs, we conducted non-parametric bootstrapping, comprising 10,000 bootstrap samples with sample lengths equaled comorbidity-specific death counts [46]. For YLDs, data simulation was also repeated 10,000 times. For each simulation, disease severity was randomly assigned based on literature-derived probabilistic distributions. Subsequently, a number within the range of corresponding severity-specific disability weights was randomly assigned [28]. YLDs were then adjusted for the presence of other comorbidities at the individual level, assuming multiplicative combined disability weights (Note A in S1 Text) [16, 26]. The 10,000 simulated values from YLLs and YLDs were then randomly paired and summed to estimate the 95% CrI of DALYs [28]. Additionally, given an ample number of prevalent cases, we sub-analyzed YLD estimates for 2008–2012 stratified by ethnicity (White, non-White, unknown), history of injection drug use (people who have ever injected drugs [PWID], non-PWID, unknown) and sex at birth (male, female).

#### Estimation of burden of cancers among PLWH, 2013–2020

With COAST data limited until 2012, we used additional data from the DTP to estimate YLLs, YLDs and DALYs associated with cancers (non-AIDS-defining only) among PLWH for 2013–2020. The DTP dataset provides demographic information on PLWH receiving ART in BC, but, unlike COAST, is not linked to healthcare utilization datasets (e.g., hospitalization, physician visit and prescription datasets). Given the absence of information on HIV-negative individuals in the DTP dataset, our analysis was restricted to PLWH. Cancers were chosen due to its adequate number of observed mortality cases among PLWH in the COAST study, where, due to the required ≥1 year follow-up, no deaths were observed in 2001 (i.e., the first calendar year of the study follow-up period). Accordingly, we employed generalized linear mixed-effects regression to model the number of deaths on age, sex, and observation year for 2002–2012, assuming a negative binomial distribution and log link (Note C in S1 Text) [47, 48]. The resulted model was then applied to the DTP dataset to estimate the annual number of cancers-specific deaths in each age-sex subgroup for 2013–2020. To estimate annual total YLLs along with the 95% CrI, we conducted parametric bootstrapping with normal approximation using standard errors associated with the projected number of deaths in each subgroup [49]. Annual age-sex-standardized YLLs per 1000 people were estimated by an identical bootstrapping process using the estimates of age-sex-specific deaths in Canada’s 2011 census population, the reference population [50]. We adapted the original GBD projections which estimated YLDs and DALYs based on the last observed YLLs-to-YLDs ratio [51, 52]. Given the fluctuation in our observed YLLs-to-YLDs ratios over the years, however, we estimated annual total and age-sex-standardized YLDs and DALYs, alongside their respective 95% CrI, based on the mean of all observed YLLs-to-YLDs ratios instead.

## Results

During 2001–2012, 8,031 PLWH and 32,124 HIV-negative controls met the eligibility criteria. The two populations were identical in age at baseline (median: 40 years, [25^th^-75^th^ percentile: 34–47]) and sex at birth (82% male), but differed slightly in the follow-up time (median: 9 years [5–12] for PLWH vs. 11 years [6–12] for HIV-negative controls) (Table D in S1 Text). Among PLWH, 38% were PWID and 38% identified as White. Fig A in S1 Text illustrates the participant selection process.

### Comparison of burden of chronic comorbidities among PLWH and HIV-negative controls, 2008–2012

**Fig 1:**
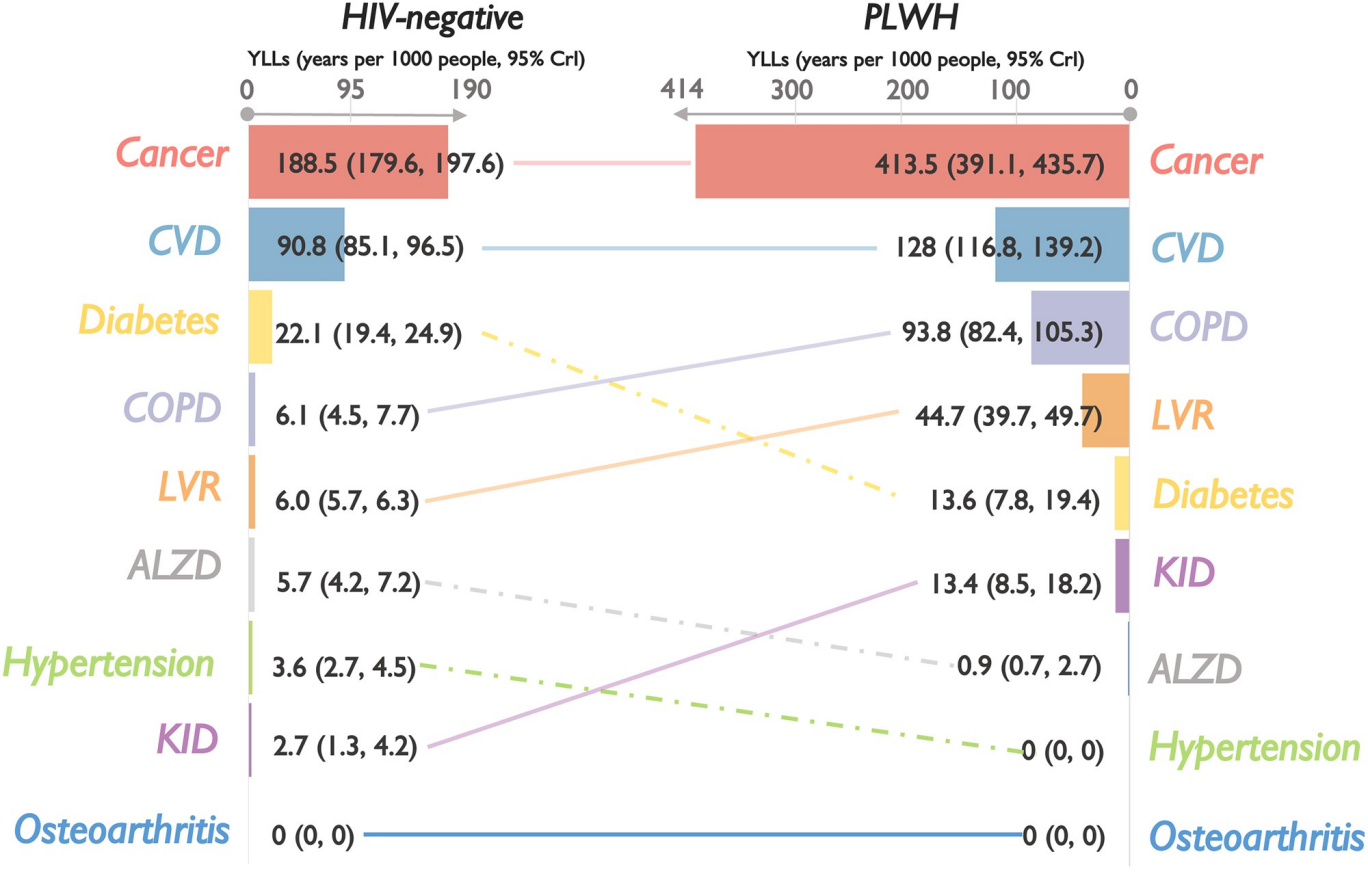
Cumulative DALYs (2008–2012) per 1000 people, with 95% credible intervals, associated with nine selected chronic comorbidities, ranked from highest to lowest, by HIV status in British Columbia, Canada. Note: PLWH: people living with HIV; DALYs: disability-adjusted life years; 95% CrI: 95% credible intervals; ALZD: Alzheimer’s and/or non-HIV-related dementia (denominator included only individuals aged 40 years and older); Cancers: non-AIDS-defining cancer; COPD: chronic obstructive pulmonary disease (denominator included only individuals aged 35 years and older); CVD: cardiovascular diseases; KID: kidney diseases; LVR: liver diseases. Horizontal scales differ between PLWH and HIV-negative controls for illustration purposes.

**Fig 2:**
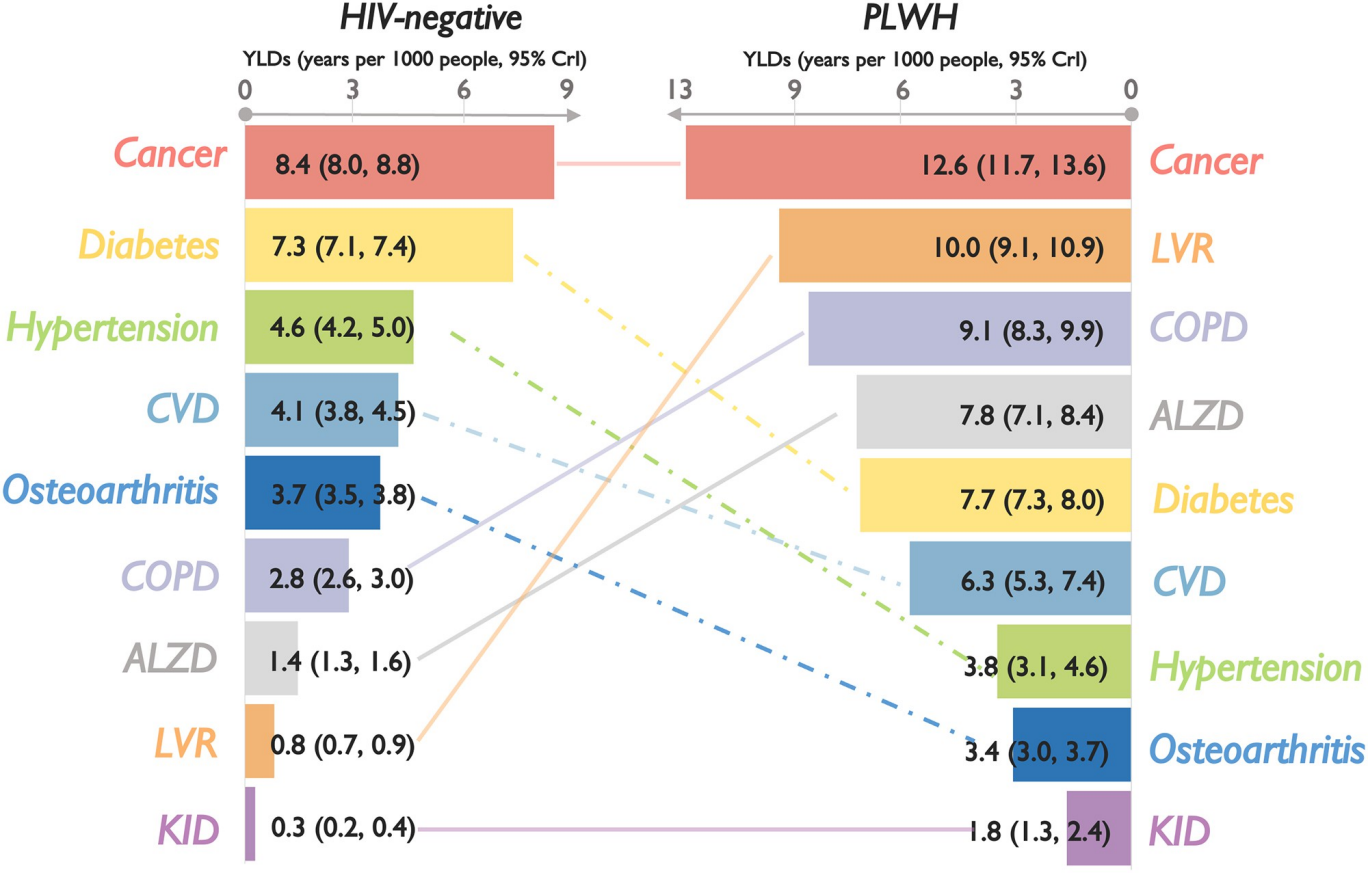
Cumulative YLLs (2008–2012) per 1000 people, with 95% credible intervals, associated with nine selected chronic comorbidities, ranked from highest to lowest, by HIV status in British Columbia, Canada. Note: PLWH: people living with HIV; YLLs: years life lost due to premature mortality; 95% CrI: 95% credible intervals; ALZD: Alzheimer’s and/or non-HIV-related dementia (denominator included only individuals aged 40 years and older); Cancers: non-AIDS-defining cancer; COPD: chronic obstructive pulmonary disease (denominator included only individuals aged 35 years and older); CVD: cardiovascular diseases; KID: kidney diseases; LVR: liver diseases. Horizontal scales differ for each graph and between PLWH and HIV-negative controls for illustration purposes.

**Fig 3:**
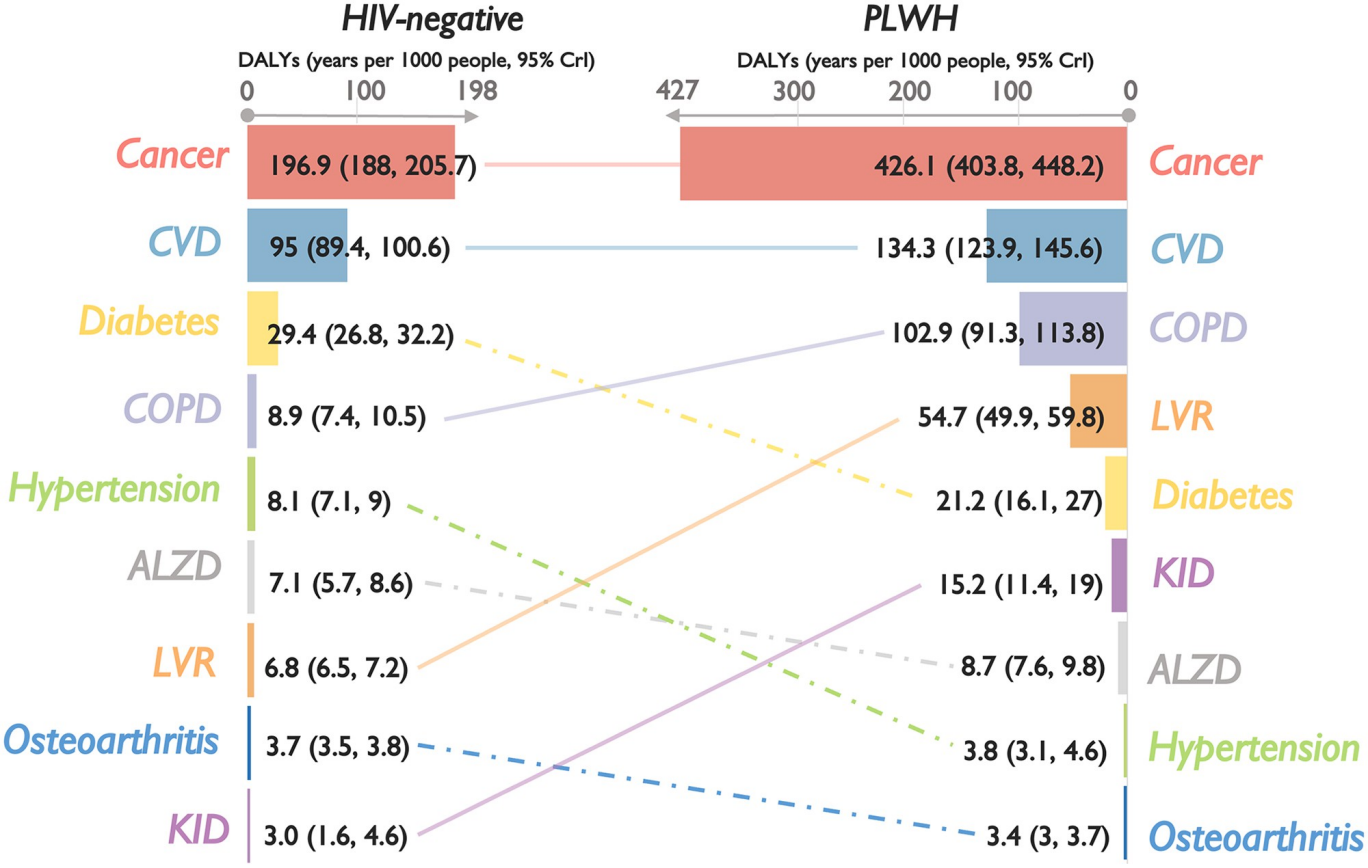
Cumulative YLDs (2008–2012) per 1000 people, with 95% credible intervals, associated with nine selected chronic comorbidities, ranked from highest to lowest, by HIV status in British Columbia, Canada. Note: PLWH: people living with HIV; YLDs: years of healthy life lost due to disability; 95% CrI: 95% credible intervals; ALZD: Alzheimer’s and/or non-HIV-related dementia (denominator included only individuals aged 40 years and older); Cancers: non-AIDS-defining cancer; COPD: chronic obstructive pulmonary disease (denominator included only individuals aged 35 years and older); CVD: cardiovascular diseases; KID: kidney diseases; LVR: liver diseases. Horizontal scales differ for each graph and between PLWH and HIV-negative controls for illustration purposes.

During 2008–2012, 7042 PLWH and 30,640 HIV-negative controls were alive, leading to 5356.5 and 10,945.7 years in estimated DALYs associated with the selected chronic comorbidities, respectively. Overall DALYs were two times higher among PLWH compared to HIV-negative controls (770.2 years/1000 people [95% CrI: 710.2, 831.6] vs. 359.0 [336.0, 382.2], respectively), with PLWH experiencing higher DALYs associated with most comorbidities, except hypertension, diabetes, and osteoarthritis (Fig 1). Except for hypertension and osteoarthritis (in both populations) and Alzheimer’s/dementia (among PLWH only), the burden of most comorbidities was driven by YLLs (Fig 2) rather than YLDs (Fig 3). Consequently, the rankings of DALYs were largely identical to those of YLLs, with cancers and CVD as predominant contributors constituting over 70% of the total estimated YLLs and DALYs in both populations. Among PLWH, COPD and liver diseases contributed the third and fourth highest YLLs and DALYs, as did diabetes and COPD among HIV-negative controls. Note, however, that DALYs related to COPD were 12 times higher among PLWH.

#### Sub-analyses of YLDs

**Fig 4:**
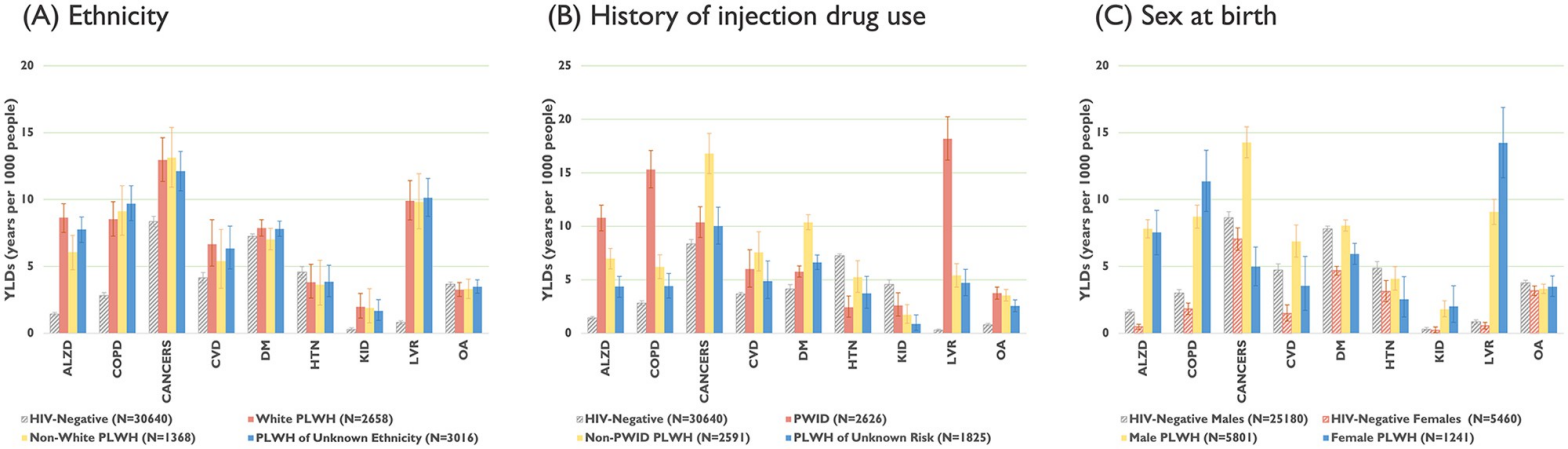
Cumulative YLDs (2008–2012) per 1000 people, with 95% credible intervals, associated with nine selected chronic comorbidities among people living with HIV in British Columbia, Canada, stratified by ethnicity (A), history of injection drug use (B) and sex at birth (C). Note: PLWH: people living with HIV; YLDs: years of healthy life lost due to disability; PWID: PLWH who have ever injected drugs; ALZD: Alzheimer’s and/or non-HIV-related dementia; CANCERS: non-AIDS-defining cancers; COPD: chronic obstructive pulmonary disease; CVD: cardiovascular diseases; DM: diabetes mellitus; HTN: hypertension; KID: kidney diseases; LVR: liver diseases; OA: osteoarthritis. Vertical scales differ for each graph for illustration purposes.

Among PLWH, during 2008–2012, YLDs associated with the selected chronic comorbidities did not vary significantly by ethnicity but varied greatly by history of injection drug use and sex at birth (Fig 4). YLDs associated with Alzheimer’s/dementia, for instance, were substantially elevated among PWID, while COPD and liver diseases were substantially elevated among PWID and female PLWH. YLDs associated with cancers, CVD and diabetes were higher among male PLWH.

### Estimation of burden of cancers among PLWH, 2013–2020

**Fig 5:**
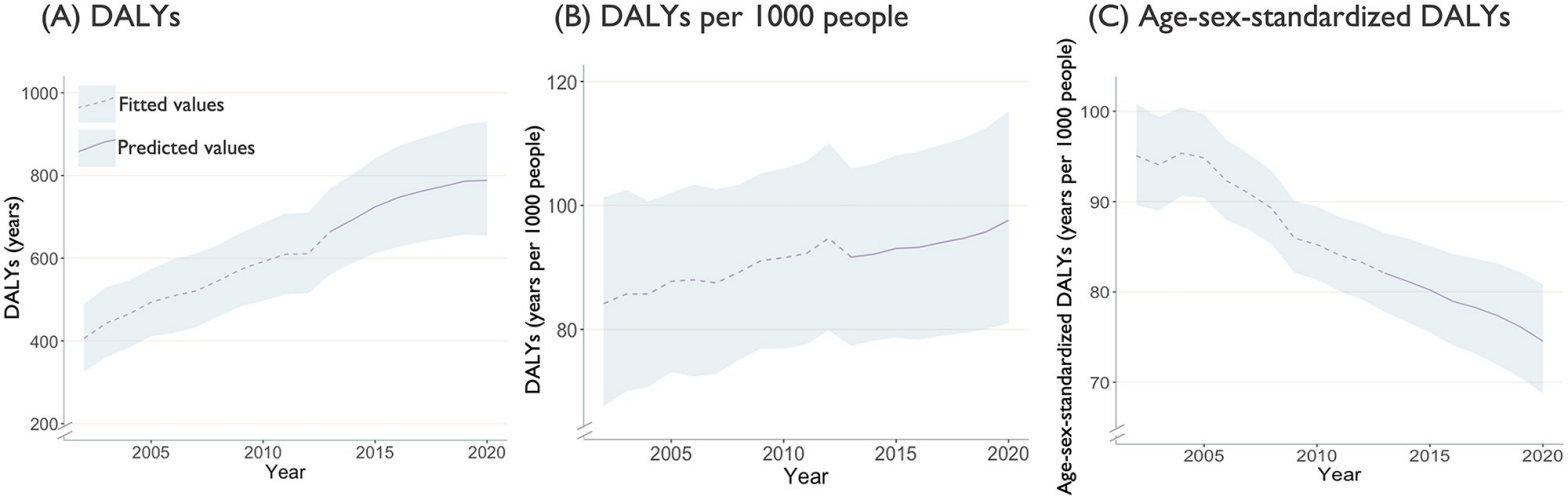
Fitted (2002–2012) and predicted (2013–2020) DALYs, with 95% credible intervals, associated with non-AIDS-defining cancers among people living with HIV in British Columbia, Canada. Note: DALYs: disability-adjusted life years. Direct age-sex-standardization was done using Canada’s 2011 census as reference population. Vertical scales differ for each graph for illustration purposes.

Of the 9601 eligible PLWH enrolled in the DTP during 2013–2020, 83% were male with median age at baseline of 47 years, (25^th^-75^th^ percentile: 38–54) and median follow-up of 8 years (5–8) (Table E in S1 Text). Population pyramids in Fig B in S1 Text illustrated changes in the age and sex distribution over the years highlighting that PLWH in BC were aging. Through 2020, we estimated an increasing trend in cancers-associated YLLs and YLDs (Fig C in S1 Text) as well as DALYs (Fig 5), overall and per 1000 PLWH. The trends in age-sex-standardized YLLs, YLDs and DALYs, however, were projected to decrease over time. In 2020, we estimated 97.6 years (95% CrI: 81.0, 115.2) in DALYs associated with cancers per 1000 people; the estimated DALYs were largely driven by YLLs with 89.3 years per 1000 people (74.1, 105.4).

## Discussion

To our knowledge, our study is the first to use DALYs to compare the burden of diseases among PLWH and age-sex-matched HIV-negative individuals. Using a large provincial administrative database which spanned over a decade, our results showed that ART-treated PLWH experienced more than double the burden of selected chronic comorbidities than their HIV-negative counterparts. In both populations, cancers and CVD contributed the most DALYs, predominantly driven by premature mortality. Diabetes and hypertension played major roles in the health burden of HIV-negative individuals, as did COPD and liver diseases in PLWH, particularly PLWH who were female or had a history of injection drug use. Additionally, among PLWH, we estimated that DALYs attributable to non-AIDS-defining cancers would continue to increase in the short term, although the age-sex standardized DALYs would decrease. Together, our findings underscore the role of different chronic comorbidities in the health burden of PLWH and the disparities of this burden compared to HIV-negative individuals, which should be considered in the prioritization of resources and interventions that address the healthcare needs of this aging population.

Consistent with observations in BC’s PLWH and HIV-negative controls, cancers and CVD contributed the highest number of DALYs in Canada and other high-resource countries [23, 53, 54]. In both study populations, YLLs contributed more than 90% of cancers-associated DALYs, congruent with findings from Japan, the Netherlands and Australia [23, 24, 54]. Similarly, the dominance of YLLs in DALYs attributable to CVD, COPD, liver, and kidney diseases have also been examined elsewhere [21–23, 55–57]. This evidence stresses the importance of strategies to prolong life and reduce preventable deaths, such as secondary and tertiary prevention focusing on early detection and minimizing adverse outcomes [58], in alleviating the burden related to these chronic comorbidities. Conversely, the burden of hypertension, which is a known risk factor for other prominent chronic comorbidities, and osteoarthritis, to which no deaths were attributed in our populations and globally [59], was driven by YLDs. In mitigating the impact of these diseases on population health, the role of primary prevention, aimed at improving awareness of risk factors and altering health risk behaviors, should thus be highlighted.

The lack of prior studies examining the disparities in DALYs across HIV status precludes any direct comparisons of our findings. However, the observed disparities, including the two-fold higher burden of chronic comorbidities among PLWH, were aligned with previous findings regarding prevalence [4, 60, 61] and mortality [62–66]. While cancers and CVD contributed significantly to the health burden of both study populations, COPD and liver diseases were also prominent among PLWH, as were diabetes and hypertension among HIV-negative controls. This discrepancy emphasizes the presence of differential health behaviors in both populations and the role of factors related to HIV infection and treatment, which should be considered when addressing the disproportionate disease burden among PLWH. First-generation nucleoside reverse transcriptase inhibitors, for example, have been associated with an increased risk of liver fibrosis [67]. Alcohol use, which is associated with a higher level of cirrhosis biomarkers in HIV-hepatitis C co-infected individuals, and smoking, a major risk factor of COPD, have also been found more prevalent among PLWH [68–71]. Additionally, among PLWH, our findings bolster the growing body of literature [72–75], and underline the merit of considering sex differences and history of injection drug use in formulating appropriate chronic disease interventions in this population.

Among BC’s PLWH, we estimated an increasing trend in DALYs associated with cancers through 2020, where annual DALY estimates per 1000 people in our population were consistently double those in Canada’s general population [76]. Concurrently, we also estimated a decreasing trend in corresponding age-sex-standardized DALYs. These trends agree with the 2012–2019 trends in the global all-cause and Canada’s cancers-associated DALYs [17, 76], and demonstrate the impact of population growth and aging in the progression of the disease burden, reinforcing the need to better understand how aging shapes a population’s healthcare needs. Particularly, the increasing DALYs represent the magnitude of the burden imposed on the healthcare system [17], which should be met with adequate infrastructure, human resources, and services. Our findings thus reaffirm present policies and interventions surrounding prevention, early screening, and management of cancers in BC, while simultaneously promoting the evolvement of these efforts, anticipating changes in population structure and needs. Future studies should project DALYs associated with other comorbidities to obtain a comprehensive outlook on the future overall health burden of PLWH, which will further elucidate optimal allocations and prioritizations of healthcare resources.

This study has some limitations. First, administrative health data, while important for clinical and public health policy-making, were not collected for research purposes. To mitigate administrative data’s susceptibility to coding errors, whenever possible, we utilized published BC Ministry of Health’s case-finding algorithms to identify prevalent comorbidity diagnoses from hospitalizations and day-surgeries, physician visits, laboratory and diagnostic procedures, and prescription drug dispensation datasets, considering BC-specific claims-related practices. Moreover, with over 10 years of follow-up and a judicious 5-year lookback window [77], we are confident in our ability to capture most prevalent cases in both populations, upholding the validity of our YLD estimations. Second, the scarcity of information on disease severity distribution is a widely recognized problem [18, 78, 79]. With administrative data lacking information on the severity of comorbidity diagnoses, we employed literature-derived disease severity distribution, using data from BC or Canada whenever available and applying the same distribution to both PLWH and HIV-negative controls. Further, the lack of empirical data on alternative severity distributions of each comorbidity or how these distributions may vary within and between the two study populations prevented us from conducting meaningful sensitivity analyses. Instead, to account for the uncertainty surrounding the severity distribution, the probabilistic resampling of disease severity status and random assignment of the corresponding disability weight were conducted for each of the 10,000 simulations. Notwithstanding, given that DALYs associated with most diseases were predominantly driven by YLLs, the observed disparities in DALYs between PLWH and HIV-negative individuals should persist despite this limitation. Third, administrative data rendered us unable to consider socioeconomic and lifestyle differences (e.g., alcohol use and smoking) in our analyses, although our sub-analyses unveiled substantial differences in YLDs of major comorbidities among PLWH by sex and history of injection drug use. Fourth, low mortality cases prevented further age- or sex-specific measurements of YLLs and DALYs, although, for YLDs, we were able to highlight key differential burdens across sex groups. Fifth, the estimated burden of non-AIDS-defining cancers among PLWH for 2013–2020 should be interpreted in light of methodological constraints and assumptions. For instance, the lack of socioeconomic and lifestyle data prevented us from conducting more sophisticated modelling to estimate cancers-specific YLLs [80]. Additionally, adapting earlier GBD projections [51, 52], cancers-specific YLDs were estimated based on the mean of all observed YLLs-to-YLDs ratios. Lastly, COAST data are currently limited to December 2012. Note, however, that during 2012–2019, the relative DALY contributions of non-communicable diseases in Canada’s general population were largely unchanged [53]. Furthermore, the methodology established in this study can be readily applied to other settings to compare the contemporary burden of chronic comorbidities between PLWH and HIV-negative controls.

In summary, our study highlights that, compared to age-sex-matched HIV-negative controls from the same geographical setting, PLWH experienced excess DALYs attributable to chronic comorbidities, with the largest contribution from premature mortality associated with non-AIDS-defining cancers and CVD. Our study also underscores the role of aging in shaping the upward trajectories of disease burden and, consequently, determining the future healthcare needs among PLWH. Overall, these findings advocate for intervention strategies that integrate primary, secondary, and tertiary prevention of chronic comorbidities into a comprehensive HIV care model, with the goals to reduce preventable deaths and morbidity and, ultimately, facilitate healthy and successful aging in this population. Factors that likely influence the observed disparities both within PLWH and between PLWH and HIV-negative individuals, such as health behaviors, sex differences, residual HIV-related inflammation, and ART-related toxicities, should also be considered in these strategies. Further research on the current and projected overall disease burden should be prioritized to ensure timely implementation of evidence-based policies and investments to ensure adequate healthcare personnel and infrastructure.

## Supporting information

S1 Text Supplemental materials. (DOCX)

## References

[1] Wing EJ. HIV and aging. Int J Infect Dis [Internet]. 2016 Dec 1 [cited 2018 Apr 24];53:61–8. Available from: https://www.sciencedirect.com/science/article/pii/S1201971216311870 doi: 10.1016/j.ijid.2016.10.004 27756678

[2] Collaboration TATC. Causes of Death in HIV-1—Infected Patients Treated with Antiretroviral Therapy, 1996–2006: Collaborative Analysis of 13 HIV Cohort Studies. Clin Infect Dis [Internet]. 2010 May 15;50(10):1387–96. Available from: doi: 10.1086/652283 20380565 PMC3157754

[3] Lima VD, Lourenço L, Yip B, Hogg RS, Phillips P, Montaner JSG. AIDS incidence and AIDS-related mortality in British Columbia, Canada, between 1981 and 2013: A retrospective study. Lancet HIV [Internet]. 2015 Mar [cited 2018 Apr 24];2(3):92–7. Available from: http://www.ncbi.nlm.nih.gov/pubmed/25780802 doi: 10.1016/S2352-3018(15)00017-X 25780802 PMC4357843

[4] Guaraldi G, Orlando G, Zona S, Menozzi M, Carli F, Garlassi E, et al. Premature Age-Related Comorbidities Among HIV-Infected Persons Compared With the General Population. Clin Infect Dis [Internet]. 2011 Dec 1 [cited 2019 Feb 8];53(11):1120–6. Available from: https://academic.oup.com/cid/article-lookup/doi/10.1093/cid/cir627 doi: 10.1093/cid/cir627 21998278

[5] Justice A, Falutz J. Aging and HIV: an evolving understanding. Curr Opin HIV AIDS [Internet]. 2014 Jul [cited 2019 Feb 4];9(4):291–3. http://www.ncbi.nlm.nih.gov/pubmed/24871091 24871091 10.1097/COH.0000000000000081 PMC4507814

[6] Mayer KH, Loo S, Crawford PM, Crane HM, Leo M, Denouden P, et al. Excess Clinical Comorbidity Among HIV-Infected Patients Accessing Primary Care in US Community Health Centers. [cited 2019 Feb 10]; https://www.ncbi.nlm.nih.gov/pmc/articles/PMC5805107/pdf/10.1177_0033354917748670.pdf 10.1177/0033354917748670 PMC5805107 29262289

[7] Wong C, Gange SJ, Moore RD, Justice AC, Buchacz K, Abraham G, et al. Multimorbidity Among Persons Living with HIV in the U.S. Clin Infect Dis. 2017;(November):1–37.

[8] Althoff KN, Smit M, Reiss P, Justice AC. HIV and ageing: improving quantity and quality of life. Curr Opin HIV AIDS [Internet]. 2016 [cited 2019 Feb 7];11(5):527–36. Available from: http://www.ncbi.nlm.nih.gov/pubmed/27367780 doi: 10.1097/COH.0000000000000305 27367780 PMC5084838

[9] BC Centre for Excellence in HIV/AIDS. HIV Monitoring Quarterly Report for British Columbia—Fourth Quarter 2020 [Internet]. Vancouver, Canada; 2020. https://stophivaids.ca/qmr/2020-Q4/#/bc

[10] Nanditha NGA, Paiero A, Tafessu HM, St-Jean M, McLinden T, Justice AC, et al. Excess burden of age-associated comorbidities among people living with HIV in British Columbia, Canada: A population-based cohort study. BMJ Open. 2021 Jan 8;11(1). 10.1136/bmjopen-2020-041734 PMC7799128 33419911

[11] Smith CJ, Ryom L, Weber R, Morlat P, Pradier C, Reiss P, et al. Trends in underlying causes of death in people with HIV from 1999 to 2011 (D:A:D): a multicohort collaboration. Lancet [Internet]. 2014 Jul 19 [cited 2019 Feb 10];384(9939):241–8. Available from: https://www-sciencedirect-com.ezproxy.library.ubc.ca/science/article/pii/S0140673614606048?via%3Dihub doi: 10.1016/S0140-6736(14)60604-8 25042234

[12] Helleberg M, Kronborg • G, Larsen • C S, Pedersen • G, Pedersen • C, Gerstoft • J, et al. Causes of death among Danish HIV patients compared with population controls in the period 1995–2008. Infection. 2012;40:627–34. doi: 10.1007/s15010-012-0293-y 22791407

[13] Lima VD, Brumme ZL, Brumme C, Sereda P, Krajden M, Wong J, et al. The Impact of Treatment as Prevention on the HIV Epidemic in British Columbia, Canada. Curr HIV/AIDS Rep [Internet]. 2020 [cited 2020 Mar 9]; Available from: doi: 10.1007/s11904-020-00482-6 32124189 PMC8797149

[14] Murray1 CJL. Quantifying the burden of disease: the technical basis for disability-adjusted life years. WHO Bull OMS. 1994;72:1994. PMC2486718 8062401

[15] Murray CJ, Lopez AD. The Global burden of Diseases: A comprehensive assessment of mortality and disability from diseases, injuries, and risk factors in 1990 and projected to 2020 EDITED BY Library of Congress Cataloging-in-Publication (CIP) Data applied for [Internet]. Boston, USA; 1996 [cited 2022 Feb 11]. https://apps.who.int/iris/bitstream/handle/10665/41864/0965546608_eng.pdf

[16] Cao B, Stevens G, Ho J, Fat DM. WHO methods and data sources for global burden of disease estimates 2000–2019. Geneva; 2020.

[17] Abbafati C, Abbas KM, Abbasi-Kangevari M, Abd-Allah F, Abdelalim A, Abdollahi M, et al. Global burden of 369 diseases and injuries in 204 countries and territories, 1990–2019: a systematic analysis for the Global Burden of Disease Study 2019. Lancet [Internet]. 2020 Oct 17 [cited 2022 Jan 20];396(10258):1204–22. Available from: http://www.thelancet.com/article/S0140673620309259/fulltext doi: 10.1016/S0140-6736(20)30925-9 33069326 PMC7567026

[18] Rommel A, von der Lippe E, Plaß D, Wengler A, Anton A, Schmidt C, et al. BURDEN 2020-Burden of disease in Germany at the national and regional level. Bundesgesundheitsblatt Gesundheitsforschung Gesundheitsschutz [Internet]. 2018 Sep 1 [cited 2022 Feb 11];61(9):1159–66. https://pubmed.ncbi.nlm.nih.gov/30083946/10.1007/s00103-018-2793-0 30083946

[19] Yoon SJ, Go DS, Park H, Jo MW, Oh IH, Kim YE. The Korean National Burden of Disease Study: from Evidence to Policy. J Korean Med Sci [Internet]. 2019 Mar 26 [cited 2022 Feb 11];34(Suppl 1). Available from: /pmc/articles/PMC6434148/ doi: 10.3346/jkms.2019.34.e89 30923492 PMC6434148

[20] Kaduka L, Muniu E, Mbui J, Oduor Owuor C, Gakunga R, Kwasa J, et al. Disability-Adjusted Life-Years Due to Stroke in Kenya. Neuroepidemiology [Internet]. 2019 Aug 1 [cited 2022 Feb 11];53(1–2):48–54. Available from: https://pubmed.ncbi.nlm.nih.gov/30986786/ doi: 10.1159/000498970 30986786

[21] Fernández de Larrea-Baz N, Morant-Ginestar C, Catalá-López F, Gènova-Maleras R, Álvarez-Martín E. Disability-adjusted Life Years Lost to Ischemic Heart Disease in Spain. Rev Esp Cardiol (Engl Ed) [Internet]. 2015 Nov [cited 2022 Feb 11];68(11):968–75. Available from: https://pubmed.ncbi.nlm.nih.gov/25887346/ doi: 10.1016/j.rec.2014.11.024 25887346

[22] Coute RA, Nathanson BH, Panchal AR, Kurz MC, Haas NL, McNally B, et al. Disability-Adjusted Life Years Following Adult Out-of-Hospital Cardiac Arrest in the United States. Circ Cardiovasc Qual Outcomes [Internet]. 2019 Mar 1 [cited 2022 Feb 11];12(3). Available from: https://pubmed.ncbi.nlm.nih.gov/30859852/ doi: 10.1161/CIRCOUTCOMES.118.004677 30859852

[23] Moon L, Garcia J, Laws P, Dunford M, On ML, Bishop K, et al. Measuring Health Loss in Australia: the Australian Burden of Disease Study. J Korean Med Sci [Internet]. 2019 Mar 26 [cited 2022 Feb 11];34(Suppl 1):2013–5. Available from: /pmc/articles/PMC6434150/ doi: 10.3346/jkms.2019.34.e61 30923485 PMC6434150

[24] Pham TM, Kubo T, Fujino Y, Ozasa K, Matsuda S, Yoshimura T. Disability-Adjusted Life Years (DALY) for Cancer in Japan in 2000. J Epidemiol [Internet]. 2011 [cited 2022 Feb 11];21(4):309. Available from: /pmc/articles/PMC3899425/ doi: 10.2188/jea.je20110017 21628841 PMC3899425

[25] Park B, Park B, Han H, Choi EJ, Kim NE, Shin Y, et al. Projection of the years of life lost, years lived with disability, and disability-adjusted life years in Korea for 2030. J Korean Med Sci [Internet]. 2019 Mar 26 [cited 2021 Mar 31];34(Suppl 1). Available from: /pmc/articles/PMC6434152/10.3346/jkms.2019.34.e92 PMC6434152 30923495

[26] Hilderink HBM, Plasmans MHD, Snijders BEP, Boshuizen HC, René Poos MJJC, van Gool CH. Accounting for multimorbidity can affect the estimation of the Burden of Disease: a comparison of approaches. Arch Public Heal [Internet]. 2016 Aug 22 [cited 2022 Jan 28];74(1). Available from: /pmc/articles/PMC4993005/10.1186/s13690-016-0147-7 PMC4993005 27551405

[27] Lin X, Bloom MS, Du Z, Hao Y. Trends in disability-adjusted life years of lung cancer among women from 2004 to 2030 in Guangzhou, China: A population-based study. Cancer Epidemiol. 2019 Dec 1;63:101586. doi: 10.1016/j.canep.2019.101586 31522131

[28] von der Lippe E, Devleesschauwer B, Gourley M, Haagsma J, Hilderink H, Porst M, et al. Reflections on key methodological decisions in national burden of disease assessments. Arch Public Heal 2020 781 [Internet]. 2020 Dec 31 [cited 2021 Sep 15];78(1):1–14. Available from: https://archpublichealth.biomedcentral.com/articles/ doi: 10.1186/s13690-020-00519-7 33384020 PMC7774238

[29] Eyawo O, Hull MW, Salters K, Samji H, Cescon A, Sereda P, et al. Cohort profile: the Comparative Outcomes And Service Utilization Trends (COAST) Study among people living with and without HIV in British Columbia, Canada. BMJ Open [Internet]. 2018 [cited 2018 Mar 26];8(1):e019115. Available from: http://www.ncbi.nlm.nih.gov/pubmed/29331972 doi: 10.1136/bmjopen-2017-019115 29331972 PMC5781099

[30] British Columbia Centre for Excellence in HIV/AIDS (BCCfE). Drug Treatment Program [Internet]. 2019 [cited 2018 Apr 24]. http://www.cfenet.ubc.ca/drug-treatment-program%0Ahttp://www.cfenet.ubc.ca/research/laboratory-program

[31] British Columbia Ministry of Health [creator] (2014). Consolidation File (MSP Registration & Premium Billing). V2. Population Data BC [publisher]. Data Extract. MOH (2014) [Internet]. [cited 2019 Feb 17]. http://www.popdata.bc.ca/data

[32] Canadian Institute for Health Information [creator] (2014). Discharge Abstract Database (Hospital Separations). V2. Population Data BC [publisher]. Data Extract. MOH (2014) [Internet]. http://www.popdata.bc.ca/data

[33] British Columbia Ministry of Health [creator] (2014). Medical Services Plan (MSP) Payment Information File. V2. Population Data BC [publisher]. Data Extract. MOH (2014). [Internet]. [cited 2019 Feb 17]. http://www.popdata.bc.ca/data

[34] British Columbia Ministry of Health [creator] (2014). Vital Events Deaths. V2. Population Data BC [publisher]. Data Extract. MOH (2014) [Internet]. http://www.popdata.bc.ca/data

[35] BC Cancer [creator] (2016). BC Cancer Registry Data. V2. Population Data BC [publisher]. Data Extract. BC Cancer (2014) [Internet]. http://www.popdata.bc.ca/data

[36] British Columbia Ministry of Health [creator] (2014). PharmaNet. V2. Population Data BC [publisher]. Data Extract. Data Stewardship Committee (2014) [Internet]. http://www.popdata.bc.ca/data

[37] Chronic Disease Information Working Group. BC Chronic Disease and Selected Procedure Case Definitions version 2017, last updated April 2019 [Internet]. British Columbia Ministry of Health; 2015. http://www.bccdc.ca/health-professionals/data-reports/chronic-disease-dashboard#Case—Definitions

[38] Nanditha NGA, Zheng G, Tafessu HM, McLinden T, Bratu A, Kopec J, et al. Disparities in multimorbidity and mortality among people living with and without HIV across British Columbia’s health regions: a population-based cohort study. Can J Public Heal [Internet]. 2021 Dec 1 [cited 2022 Mar 10];112(6):1030–41. Available from: https://link.springer.com/article/ doi: 10.17269/s41997-021-00525-4 34462891 PMC8651938

[39] Salomon JA, Vos T, Hogan DR, Gagnon M, Naghavi M, Mokdad A, et al. Common values in assessing health outcomes from disease and injury: disability weights measurement study for the Global Burden of Disease Study 2010. Lancet (London, England) [Internet]. 2012 [cited 2022 Jan 26];380(9859):2129–43. Available from: https://pubmed.ncbi.nlm.nih.gov/23245605/ doi: 10.1016/S0140-6736(12)61680-8 23245605 PMC10782811

[40] Salomon JA, Haagsma JA, Davis A, de Noordhout CM, Polinder S, Havelaar AH, et al. Disability weights for the Global Burden of Disease 2013 study. Lancet Glob Heal [Internet]. 2015 Nov 1 [cited 2022 Jan 26];3(11):e712–23. Available from: http://www.thelancet.com/article/S2214109X15000698/fulltext doi: 10.1016/S2214-109X(15)00069-8 26475018

[41] Global Burden of Disease Collaborative Network. Global Burden of Disease Study 2019 (GBD 2019) Reference Life Table [Internet]. Seattle, United States of America; 2020. http://ghdx.healthdata.org/gbd-2019

[42] Kendall K, George M. Kruskal-Wallis Test. Concise Encycl Stat [Internet]. 2008 Feb 17 [cited 2022 Mar 3];288–90. https://link.springer.com/referenceworkentry/ doi: 10.1007/978-0-387-32833-1_216

[43] Durand M, Chartrand-Lefebvre C, Baril JG, Trottier S, Trottier B, Harris M, et al. The Canadian HIV and aging cohort study—determinants of increased risk of cardiovascular diseases in HIV-infected individuals: Rationale and study protocol. BMC Infect Dis [Internet]. 2017 [cited 2019 Feb 10];17(1). Available from: https://www.ncbi.nlm.nih.gov/pmc/articles/PMC5594495/pdf/12879_2017_Article_2692.pdf doi: 10.1186/s12879-017-2692-2 28893184 PMC5594495

[44] de Vocht F, Higgerson J, Oliver K, Verma A. Incorporating uncertainty in aggregate burden of disease measures: an example of DALYs-averted by a smoking cessation campaign in the UK. J Epidemiol Community Heal [Internet]. 2011 Sep 1 [cited 2022 Jan 28];65(9):751–6. Available from: https://jech.bmj.com/content/65/9/751 doi: 10.1136/jech.2010.119842 21097808

[45] Hespanhol L, Vallio CS, Costa LM, Saragiotto BT. Understanding and interpreting confidence and credible intervals around effect estimates. Brazilian J Phys Ther [Internet]. 2019 Jul 1 [cited 2022 Jan 7];23(4):290. Available from: /pmc/articles/PMC6630113/ doi: 10.1016/j.bjpt.2018.12.006 30638956 PMC6630113

[46] Wagner RG, Ibinda F, Tollman S, Lindholm L, Newton CR, Bertram MY. Differing Methods and Definitions Influence DALY estimates: Using Population-Based Data to Calculate the Burden of Convulsive Epilepsy in Rural South Africa. PLoS One [Internet]. 2015 Dec 1 [cited 2022 Mar 11];10(12). Available from: /pmc/articles/PMC4689490/ doi: 10.1371/journal.pone.0145300 26697856 PMC4689490

[47] Delwarde A, Denuit M, Partrat C. Negative binomial version of the Lee-Carter model for mortality forecasting. Appl Stoch Model Bus Ind. 2007 Sep;23(5):385–401.

[48] Coffie E. A comparison of Poisson or Negative Binomial Regression and Lee-Carter Models of forecasting Norwegian male mortality. University of Oslo; 2015.

[49] Guzman-Castillo M, Ahmadi-Abhari S, Bandosz P, Capewell S, Steptoe A, Singh-Manoux A, et al. Forecasted trends in disability and life expectancy in England and Wales up to 2025: a modelling study. Lancet Public Heal [Internet]. 2017 Jul 1 [cited 2021 Aug 3];2(7):e307. Available from: /pmc/articles/PMC5500313/ doi: 10.1016/S2468-2667(17)30091-9 28736759 PMC5500313

[50] Statistics Canada. 2011 Census of Population [Internet]. Ottawa; 2011. https://www12.statcan.gc.ca/census-recensement/2011/dp-pd/tbt-tt/Rp-eng.cfm?TABID=4&LANG=E&A=R&APATH=3&DETAIL=0&DIM=0&FL=A&FREE=0&GC=01&GL=-1&GID=1036971&GK=1&GRP=1&O=D&PID=101998&PRID=10&PTYPE=101955&S=0&SHOWALL=0&SUB=0&Temporal=2011&THEME=88&VID=0&VNAME

[51] Mathers CD, Loncar D. Projections of Global Mortality and Burden of Disease from 2002 to 2030. PLoS Med [Internet]. 2006 Nov [cited 2021 Aug 4];3(11):2011–30. Available from: /pmc/articles/PMC1664601/ doi: 10.1371/journal.pmed.0030442 17132052 PMC1664601

[52] Murray CJL, Lopez AD. Alternative projections of mortality and disability by cause 1990–2020: Global Burden of Disease Study. Lancet. 1997 May 24;349(9064):1498–504. doi: 10.1016/S0140-6736(96)07492-2 9167458

[53] Institute for Health Metrics and Evaluation (IHME). GBD Compare [Internet]. Seattle, United States of America; 2020 [cited 2022 Feb 22]. http://vizhub.healthdata.org/gbd-compare

[54] Hilderink HBM, Plasmans MHD, Poos MJJC, Eysink PED, Gijsen R. Dutch DALYs, current and future burden of disease in the Netherlands. Arch Public Heal [Internet]. 2020 Sep 22 [cited 2022 Feb 19];78(1). Available from: /pmc/articles/PMC7510132/ doi: 10.1186/s13690-020-00461-8 32983448 PMC7510132

[55] Soriano JB, Kendrick PJ, Paulson KR, Gupta V, Abrams EM, Adedoyin RA, et al. Prevalence and attributable health burden of chronic respiratory diseases, 1990–2017: a systematic analysis for the Global Burden of Disease Study 2017. Lancet Respir Med [Internet]. 2020 Jun 1 [cited 2022 Feb 20];8(6):585. Available from: /pmc/articles/PMC7284317/ doi: 10.1016/S2213-2600(20)30105-3 32526187 PMC7284317

[56] Paik JM, Golabi P, Younossi Y, Saleh N, Nhyira A, Younossi ZM. The Growing Burden of Disability Related to Chronic Liver Disease in the United States: Data From the Global Burden of Disease Study 2007‐2017. Hepatol Commun [Internet]. 2021 May 1 [cited 2022 Feb 20];5(5):749. Available from: /pmc/articles/PMC8122384/ doi: 10.1002/hep4.1673 34027266 PMC8122384

[57] Bowe B, Xie Y, Li T, Mokdad AH, Xian H, Yan Y, et al. Changes in the US Burden of Chronic Kidney Disease From 2002 to 2016: An Analysis of the Global Burden of Disease Study. JAMA Netw Open [Internet]. 2018 Nov 2 [cited 2022 Feb 20];1(7):e184412. Available from: /pmc/articles/PMC6324659/ doi: 10.1001/jamanetworkopen.2018.4412 30646390 PMC6324659

[58] Kisling LA, Das JM. Prevention Strategies. StatPearls Publ [Internet]. 2021 May 9 [cited 2022 Feb 21]; Available from: https://www.ncbi.nlm.nih.gov/books/NBK537222/

[59] Safiri S, Kolahi AA, Smith E, Hill C, Bettampadi D, Mansournia MA, et al. Global, regional and national burden of osteoarthritis 1990–2017: a systematic analysis of the Global Burden of Disease Study 2017. Ann Rheum Dis [Internet]. 2020 Jun 1 [cited 2022 Feb 20];79(6):819–28. Available from: https://ard.bmj.com/content/79/6/819 doi: 10.1136/annrheumdis-2019-216515 32398285

[60] Bekker LG, Montaner J, Ramos C, Sherer R, Celletti F, Cutler B, et al. IAPAC Guidelines for Optimizing the HIV Care Continuum for Adults and Adolescents. J Int Assoc Provid AIDS Care [Internet]. 2015 Nov 2 [cited 2020 Nov 1];14(1_suppl):S3–34. Available from: http://journals.sagepub.com/doi/ doi: 10.1177/2325957415613442 26527218

[61] Rasmussen LD, May MT, Kronborg G, Larsen CS, Pedersen C, Gerstoft J, et al. Time trends for risk of severe age-related diseases in individuals with and without HIV infection in Denmark: A nationwide population-based cohort study. Lancet HIV [Internet]. 2015 [cited 2019 Jan 27];2(7):e288–98. Available from: www.thelancet.com/ doi: 10.1016/S2352-3018(15)00077-6 26423253

[62] Eyawo O, Franco-Villalobos C, Hull MW, Nohpal A, Samji H, Sereda P, et al. Changes in mortality rates and causes of death in a population-based cohort of persons living with and without HIV from 1996 to 2012. BMC Infect Dis. 2017;17(1):1–15. 28241797 10.1186/s12879-017-2254-7 PMC5329918

[63] Aldaz P, Moreno-Iribas C, Egüés N, Irisarri F, Floristan Y, Sola-Boneta J, et al. Mortality by causes in HIV-infected adults: comparison with the general population. BMC Public Health [Internet]. 2011 Dec 11 [cited 2020 Jan 31];11(1):300. Available from: http://bmcpublichealth.biomedcentral.com/articles/ doi: 10.1186/1471-2458-11-300 21569323 PMC3112125

[64] Coghill AE, Pfeiffer RM, Shiels MS, Engels EA. Excess Mortality among HIV-infected Individuals with Cancer in the United States. Cancer Epidemiol Biomarkers Prev [Internet]. 2017 Jul 1 [cited 2022 Feb 21];26(7):1027. Available from: /pmc/articles/PMC5500417/ doi: 10.1158/1055-9965.EPI-16-0964 28619832 PMC5500417

[65] Martínez E, Milinkovic A, Buira E, De Lazzari E, Leó A, Larrousse M, et al. Incidence and causes of death in HIV-infected persons receiving highly active antiretroviral therapy compared with estimates for the general population of similar age and from the same geographical area. 10.1111/j.1468-1293.2007.00468.x 17461853

[66] Whiteside YO, Selik R, An Q, Huang T, Karch D, Hernandez AL, et al. Comparison of Rates of Death Having any Death-Certificate Mention of Heart, Kidney, or Liver Disease Among Persons Diagnosed with HIV Infection with those in the General US Population, 2009–2011. Open AIDS J [Internet]. 2015 Mar 6 [cited 2022 Feb 21];9(1):14. Available from: /pmc/articles/PMC4353126/10.2174/1874613601509010014 PMC4353126 25767634

[67] Bakasis AD, Androutsakos T. Liver Fibrosis during Antiretroviral Treatment in HIV-Infected Individuals. Truth or Tale? Cells 2021, Vol 10, Page 1212 [Internet]. 2021 May 15 [cited 2022 Feb 21];10(5):1212. Available from: https://www.mdpi.com/2073-4409/10/5/1212/htm doi: 10.3390/cells10051212 34063534 PMC8156893

[68] Williams EC, Hahn JA, Saitz R, Bryant K, Lira MC, Samet JH. Alcohol Use and Human Immunodeficiency Virus (HIV) Infection: Current Knowledge, Implications, and Future Directions. Alcohol Clin Exp Res [Internet]. 2016 [cited 2019 Apr 22];40(10):2056–72. Available from: http://www.ncbi.nlm.nih.gov/pubmed/27696523 27696523 10.1111/acer.13204 PMC5119641

[69] Tsui JI, Cheng DM, Libman H, Bridden C, Saitz R, Samet JH. Risky alcohol use and serum aminotransferase levels in HIV-infected adults with and without hepatitis C. J Stud Alcohol Drugs [Internet]. 2013 Mar [cited 2019 Apr 22];74(2):266–70. Available from: http://www.ncbi.nlm.nih.gov/pubmed/23384374 doi: 10.15288/jsad.2013.74.266 23384374 PMC3568165

[70] Bien-Gund CH, Choi GH, Mashas A, Shaw PA, Miller M, Gross R, et al. Persistent Disparities in Smoking Rates Among PLWH Compared to the General Population in Philadelphia, 2009–2014. AIDS Behav [Internet]. 2021 Jan 1 [cited 2022 Feb 21];25(1):148–53. Available from: https://pubmed.ncbi.nlm.nih.gov/32591983/32591983 10.1007/s10461-020-02952-9 PMC7762737

[71] De Socio GV, Pasqualini M, Ricci E, Maggi P, Orofino G, Squillace N, et al. Smoking habits in HIV-infected people compared with the general population in Italy: a cross-sectional study. BMC Public Health [Internet]. 2020 May 20 [cited 2022 Feb 21];20(1). Available from: https://pubmed.ncbi.nlm.nih.gov/32434482/10.1186/s12889-020-08862-8 PMC7238525 32434482

[72] Palella FJ, Hart R, Armon C, Tedaldi E, Yangco B, Novak R, et al. Non-AIDS comorbidity burden differs by sex, race, and insurance type in aging adults in HIV care. AIDS [Internet]. 2019 Dec 1 [cited 2020 Dec 2];33(15):2327–35. Available from: https://pubmed.ncbi.nlm.nih.gov/31764098/31764098 10.1097/QAD.0000000000002349 PMC12329769

[73] Frazier EL, Sutton MY, Tie Y, Fagan J, Fanfair RN. Differences by Sex in Cardiovascular Comorbid Conditions Among Older Adults (Aged 50–64 or ≥65 Years) Receiving Care for Human Immunodeficiency Virus. Clin Infect Dis [Internet]. 2019 Nov 27 [cited 2020 Dec 2];69(12):2091–100. Available from: https://academic.oup.com/cid/article/69/12/2091/5485460 doi: 10.1093/cid/ciz126 31051034

[74] Hileman CO, McComsey GA. The opioid epidemic: impact on inflammation and cardiovascular disease risk in HIV. Curr HIV/AIDS Rep [Internet]. 2019 Oct 1 [cited 2022 Feb 21];16(5):381. Available from: /pmc/articles/PMC6814576/ doi: 10.1007/s11904-019-00463-4 31473903 PMC6814576

[75] Lesko CR, Moore RD, Tong W, Lau B. Association of injection drug use with incidence of HIV-associated non-AIDS-related morbidity by age, 1995–2014. AIDS [Internet]. 2016 [cited 2022 Feb 21];30(9):1447. Available from: /pmc/articles/PMC4864121/10.1097/QAD.0000000000001087 PMC4864121 26990627

[76] Institute for Health Metrics and Evaluation (IHME). GBD Results Tool [Internet]. Seattle, United States of America; 2020 [cited 2022 Feb 22]. http://ghdx.healthdata.org/gbd-results-tool

[77] Nanditha NGA, Dong X, McLinden T, Sereda P, Kopec J, Hogg RS, et al. The impact of lookback windows on the prevalence and incidence of chronic diseases among people living with HIV: an exploration in administrative health data in Canada. BMC Med Res Methodol [Internet]. 2022 Dec 1 [cited 2022 Feb 16];22(1):1–11. Available from: https://link.springer.com/articles/ doi: 10.1186/s12874-021-01448-x 34991473 PMC8734246

[78] Burstein R, Fleming T, Haagsma J, Salomon JA, Vos T, Murray CJL. Estimating distributions of health state severity for the global burden of disease study. Popul Health Metr [Internet]. 2015 Nov 18 [cited 2021 May 3];13(1):31. Available from: http://pophealthmetrics.biomedcentral.com/articles/ doi: 10.1186/s12963-015-0064-y 26582970 PMC4650517

[79] Ock M, Jo MW, Gong YH, Lee HJ, Lee J, Sim CS. Estimating the severity distribution of disease in South Korea using EQ-5D-3L: a cross-sectional study. BMC Public Health [Internet]. 2016 [cited 2022 Feb 16];16(1). Available from: /pmc/articles/PMC4782385/ doi: 10.1186/s12889-016-2904-5 26956897 PMC4782385

[80] Foreman KJ, Marquez N, Dolgert A, Fukutaki K, Fullman N, McGaughey M, et al. Forecasting life expectancy, years of life lost, and all-cause and cause-specific mortality for 250 causes of death: reference and alternative scenarios for 2016–40 for 195 countries and territories. Lancet [Internet]. 2018 Nov 10 [cited 2021 Mar 31];392(10159):2052–90. Available from: 10.1016/S0140-6736(18)31694-5 30340847 PMC6227505

