# Supplementary material for: Disability-adjusted life years associated with chronic comorbidities among people living with and without HIV: Estimating health burden in British Columbia, Canada
Source: PLOS Glob Public Health. 2022 Oct 14;2(10):e0001138. doi: 10.1371/journal.pgph.0001138 (PMC10021313; doi:10.1371/journal.pgph.0001138)
Supplement: S1 Text — (DOCX) [file pgph.0001138.s001.docx]

***Supplemental Materials***

Disability-adjusted life years associated with chronic comorbidities among people living with and without HIV: Estimating health burden in British Columbia, Canada

Ni Gusti Ayu Nanditha,^1,2^ Jielin Zhu,^1^ Lu Wang,^1^ Jacek Kopec,^3,4^ Robert S. Hogg,^1,5^, Julio S. G. Montaner^1,2^, Viviane D. Lima^1,2^

^1^ British Columbia Centre for Excellence in HIV/AIDS, Vancouver, Canada

^2^ Department of Medicine, Faculty of Medicine, University of British Columbia, Vancouver, Canada

^3^ Arthritis Research Canada, Richmond, BC, Canada

^4^ School of Population and Public Health, University of British Columbia, Vancouver, BC, Canada

^5^ Faculty of Health Sciences, Simon Fraser University, Burnaby, BC, Canada

***Corresponding author**: Viviane D. Lima

BC Centre for Excellence in HIV/AIDS
608-1081 Burrard Street
Vancouver, BC | Canada V6Z 1Y6
Tel: 1-604-806-8796

E-mail: [vlima@bccfe.ca](mailto:vlima@bccfe.ca)

**Table of Contents**

Page 3 **Note A.** Adjustment of YLD by the presence of other comorbidities.

Page 3 **Note B.** Explanation on the matching procedure to select HIV-negative control population.

Page 4 **Note C.** Model definition for estimation of burden of cancers among PLWH, 2013-2020.

Page 5 **Table A.** List of selected chronic comorbidities and relevant case-finding algorithms.

Page 12 **Table B.** Disability weight and disease severity distribution associated with selected chronic comorbidities.

Page 17 **Table C.** Reference life table or theoretical minimum risk life table (TMRLT) used in GBD 2019 to calculate YLL.

Page 18 **Table D.** Descriptive baseline characteristics of a 1:4 matched analytical sample of PLWH and HIV-negative individuals in British Columba, Canada.

Page 19 **Table E.** Descriptive baseline characteristics of an analytical sample of PLWH in British Columba, Canada receiving ART through the Drug Treatment Program during 2013-2020.

Page 20 **Fig A.** Flowchart outlining derivation of final 1:4 matched analytical sample of 8,031 PLWH and 32,124 HIV-negative individuals in British Columba, Canada (2001-2012).

Page 21 **Fig B.** Population pyramids depicting age and sex distribution among ART-treated people living with HIV in British Columbia, Canada for the year 2002 (A), 2012 (B) and 2020 (C).

Page 22 **Fig C.** Fitted (2002-2012) and predicted (2013-2020) overall YLLs (A), YLDs (B) and DALYs (C) in years, with 95% credible intervals, associated with non-AIDS-defining cancers among people living with HIV in British Columbia, Canada.

Page 23 **References**

**Note A.** Adjustment of YLD by the presence of other comorbidities.

For each individual, years of healthy life lost due to disability (YLD) was adjusted by the presence of other comorbidities by assuming the multiplicative approach of the effect of each disease (1,2):

$$DW combined=1-\left[ \left( 1-{DW}_{1} \right)\times\left( 1-{DW}_{2} \right)\times\cdots\times\left( 1-{DW}_{k} \right) \right],$$

$$DW combined=1-\prod_{i=1}^{k} \left( 1-{DW}_{i} \right),$$

where $DW combined$ is an individual’s combined disability weight, and ${DW}_{1}$, ${DW}_{2}$ and ${DW}_{k}$ are the unadjusted disability weight for *k* number of individual comorbidities. Adjusted disability weights for each comorbidity were derived by multiplying the combined disability weight with the ratio of the comorbidity’s unadjusted disability weight to the sum of unadjusted disability weight of all comorbidities.

**Note B.** Explanation on the matching procedure to select HIV-negative control population.

The chosen 1:4 matching ratio was commonly used in published matched-cohort studies (80). Increasing a matching ratio from 1:1 to 1:4 have been reported to increase a study’s power to detect treatment effects, with a 1:4 matching ratio resulting in power estimates that were only slightly below those demonstrated by confounder-adjusted regression models (81). People living with HIV (PLWH) without sufficient matched controls were excluded; therefore, restricting the number of required matched controls to no more than four controls maximized the number of PLWH included in our study. Of note, while we matched on birth year and sex at birth, we opted against matching on health authority of residence (i.e., the only other sociodemographic covariate available for the control population) given that individuals may relocate to different regions within British Columbia throughout the study period. We were also unable to match by race or ethnicity as this information was not available for the HIV-negative control population.

**Note C.** Model definition for estimation of burden of cancers among PLWH, 2013-2020.

A generalized linear mixed-effects regression model was constructed using PROC GLIMMIX in SAS software version 9.4 (SAS, Cary, North Carolina, United States):

- Response distribution: Negative Binomial
- Link function: Log
- Estimation technique: Residual Pseudo-Likelihood
- Degrees of Freedom Method: Satterthwaite
- Optimization technique: None
- Covariance structure: Unstructured
- Response variable: count of cancers-specific death
- Covariates: age (continuous, time-varying); sex (categorical, time-fixed), observation year (continuous, time-varying)
- Offset variable: log person-years

**Table A.** List of selected chronic comorbidities and relevant case-finding algorithms.

| **Chronic Comorbidities** | | **Case Definitions** | **Diagnostic, Procedure, and/or Drug Codes** | **References** |
| --- | --- | --- | --- | --- |
| Alzheimer’s and non-HIV-related dementia (ALZD) | | Applicable to persons aged 40 years and older:  1 hospitalization with an ALZD diagnostic code   OR   3 physician visits, at least 30 days apart, in 2 years with ALZD diagnostic code(s)  OR  1 ALZD prescription drug | International Classification of Diseases Ninth Revision (ICD-9): 046.1, 290, 294, 331, 331.0, 331.1, 331.5  International Classification of Diseases Tenth Revision (ICD-10): G30, F00, F01, F02, F03  Drug Identification Numbers (DIN):  2232043, 2232044, 2242115, 2242116, 2242117, 2242118, 2244298, 2244299, 2244300, 2244302, 2245240, 2260638, 2266717, 2266725, 2266733, 2269457, 2269465, 2293021, 2293048, 2293056, 2295229, 2295237, 2295245, 2302845, 2302853, 2305984, 2305992, 2306018, 2306026, 2306034, 2306042, 2306050, 2306069, 2307685, 2307693, 2307707, 2307715, 2308169, 2308177, 2308185, 2308193, 2311283, 2311291, 2311305, 2311313, 2312492, 2312506, 2312514, 2312522, 2316943, 2316951, 2316978, 2320908, 2321130, 2321599, 2321602, 2322331, 2322358, 2324059, 2324067, 2324563, 2324571, 2324598, 2324601, 2328666, 2328682, 2332809, 2332817, 2332825, 2332833, 2333376, 2333384, 2333392, 2336715, 2336723, 2336731, 2336758, 2339439, 2339447, 2339455, 2340607, 2340615, 2344807, 2348950, 2349116, 2359472, 2359480, 2362260, 2362279, 2366487, 2367688, 2367696, 2375532, 2375729, 2375737, 2375745, 2375753, 2376334, 2377950, 2377969, 2377977, 2381508, 2381516, 2382830, 2386003, 2386011, 2386038, 2386046, 2392283, 2392291, 2392305, 2395584, 2395592, 2397595, 2397609, 2397617, 2397625, 2398370, 2398389, 2398397, 2398885, 2398893, 2400561, 2400588, 2401614, 2401622, 2401630, 2401649, 2402092, 2402106, 2402645, 2402653, 2404419, 2404427, 2406985, 2406993, 2407000, 2407019, 2408600, 2408619, 2409887, 2409895, 2412853, 2412861, 2412918, 2412934, 2413671, 2413698, 2416417, 2416425, 2416573, 2416581, 2416603, 2416948, 2416956, 2416999, 2417006, 2417014, 2417022, 2419238, 2419246, 2419254, 2419866, 2419874, 2420597, 2420600, 2420821, 2420848, 2420856, 2421364, 2421453, 2421461, 2423413, 2423421, 2425157, 2425165, 2425173, 2425343, 2425351, 2425742, 2426293, 2426307, 2426943, 2426951, 2427273, 2427567, 2427575, 2427583, 2427591, 2428482, 2428490, 2430371, 2432684, 2432692, 2432803, 2439557, 2439565, 2443015, 2443023, 2443031, 2443082, 2446049, 2446669, 2446677, 2447002, 2447010 | (3) |
| Cardiovascular diseases (CVD) | Acute myocardial infarction (AMI) | 1 hospitalization with an AMI diagnostic code | ICD-9: 410  ICD-10: I21 | (3) |
|  | Congestive heart failure (CHF) | 1 hospitalization with a CHF diagnostic code   OR   2 physician visits in 1 year with CHF diagnostic code(s) | ICD-9: 428  ICD-10: I50 | (3) |
|  | Ischaemic heart disease (IHD) | Applicable to persons aged 20 years and older:  2 physician visits with Angina ICD-9 code 413 plus 1 prescription  in 1 year  OR  1 specialist visit with Angina ICD-9 code 413 plus one prescription in 1 year  OR  2 physician visits with two ICD9 codes 410, 411, 412, 413, 414 in 1 year  OR  1 CABG, PCI/PCTA procedure code  OR  1 hospitalization with any IHD code(s) | ICD-9: 410, 411, 412, 413, 414  ICD-10: I20, I21, I22, I23, I24, I25  Canadian Classification of Health Interventions (CCI):  *CABG: 1IJ57LA, 1IJ57VS, 1IJ76  *PCI/PTCA: 1U50 ,1IJ57G  Canadian Classification of Diagnostic, Therapeutic, and Surgical Procedures (CCP):  *CABG:  4811, 4812, 4813, 4814, 4815, 4816, 4817, 4819  *PCI/PTCA:  4802, 4803  **Note: CABG: coronary artery bypass surgery; PCI/PTCA:* *percutaneous coronary intervention/* *Percutaneous transluminal coronary angioplasty* | (3) |
|  | Cerebrovascular accident (stroke/CVA) | Applicable to persons aged 20 years and older:  1 hospitalization with a CVA diagnostic code  *Note: Cases occurring on the same day as a traumatic brain injury event are excluded.* | ICD-9: 362.3, 430, 431, 433.x1, 434, 435, 436  ICD-10: H34.1, I60, I61, I63, I64  *Exclusions: any traumatic brain injury*  ICD-9: 800, 801, 802, 803, 804, 850, 851, 852, 853, 854, V57.x  ICD-10: S02.x, S02.5, S06.x, Z50.x | (3) |
|  | Transient Ischemic Attack (mini stroke/TIA) | Looking at persons aged 20 and older:  1 hospitalization with a TIA diagnostic code   *Note: Cases occurring on the same day as a traumatic brain injury event are excluded. See exclusion codes for CVA above.* | ICD-9: 435  ICD-10: H34.0, G45.0, G45.1, G45.2, G45.3, G45.8, G45.9 | (3) |
| Chronic obstructive pulmonary disease (COPD) | | Looking at persons aged 35 and older:  1 hospitalization with a COPD diagnostic code   OR  2 physician visits in 1 year with COPD diagnostic code(s) | ICD-9: 491, 492, 496  ICD-10: J41, J42, J43, J44 | (4) |
| Diabetes mellitus (DM) | | 1 hospitalization with a DM diagnostic code   OR  2 physician visits in 1 year with DM diagnostic code(s)  OR  2 or more insulin prescriptions in 1 year  OR  2 or more oral antihyperglycemic (not including metformin) prescriptions in 1 year  OR  1 insulin and 1 oral antihyperglycemic (including metformin) in 1 year  OR  2 metformin prescriptions and 1 physician visit with diabetes code(s) in 1 year.  *Note: Cases of suspected gestational diabetes in women aged 10-54 are not included by excluding hospitalizations, physician claims or prescriptions within the time period 120 days preceding or 180 days after hospital records containing birth-related diagnostic codes (see gestational diabetes exclusion codes).* | ICD-9: 250  ICD-10: E10, E11, E12, E13, E14  Drug Identification Numbers (DIN): 5894, 6009, 12556, 12564, 12599, 12602, 12610, 13730, 13889, 15598, 21350, 21849, 24708, 24716, 93033, 156663, 156728, 178543, 209872, 209937, 237000, 244449, 271330, 274119, 274127, 275409, 275417, 275425, 312711, 312762, 314552, 377937, 399302, 420336, 430986, 431168, 446564, 446572, 446580, 446599, 446602, 446610, 454753, 480290, 480304, 513644, 514535, 514551, 539201, 539244, 542911, 542938, 542946, 546348, 552259, 552267, 552275, 554820, 586714, 586773, 587737, 612162, 612170, 612189, 612197, 612200, 612219, 612227, 612235, 612243, 612251, 612278, 612359, 614416, 628301, 632651, 632678, 632686, 632694, 644358, 646148, 648094, 650935, 720933, 720941, 723789, 733075, 765996, 773654, 795879, 808733, 808741, 889091, 889105, 889113, 889121, 999717, 1900927, 1900935, 1913654, 1913662, 1913670, 1913689, 1934066, 1934074, 1934082, 1934090, 1934104, 1934112, 1959212, 1959220, 1959239, 1959352, 1959360, 1962639, 1962647, 1962655, 1962663, 1985930, 1985949, 1985957, 1985965, 1985973, 1985981, 1986085, 1986791, 1986805, 1986813, 1986821, 1987534, 1987542, 1987828, 1987836, 2020734, 2020742, 2022230, 2022249, 2024217, 2024225, 2024233, 2024241, 2024268, 2024276, 2024284, 2024292, 2024306, 2024314, 2024322, 2024403, 2024446, 2025248, 2025256, 2045710, 2084341, 2085887, 2099233, 2147521, 2147548, 2148765, 2155850, 2162822, 2162849, 2167786, 2188902, 2190885, 2190893, 2220628, 2223562, 2224550, 2224569, 2224771, 2224798, 2226804, 2226812, 2228920, 2228939, 2229516, 2229517, 2229519, 2229595, 2229596, 2229656, 2229704, 2229705, 2229785, 2229994, 2230026, 2230027, 2230036, 2230037, 2230443, 2230444, 2230475, 2230670, 2230671, 2231058, 2231095, 2231096, 2231389, 2233562, 2233999, 2234513, 2234514, 2236543, 2236548, 2236733, 2236734, 2236985, 2236986, 2237531, 2238103, 2238469, 2238470, 2238471, 2238698, 2238827, 2239081, 2239214, 2239474, 2239475, 2239476, 2239924, 2239925, 2239926, 2240294, 2240295, 2240297, 2241111, 2241112, 2241113, 2241114, 2241283, 2241310, 2242095, 2242096, 2242572, 2242573, 2242574, 2242589, 2242726, 2242783, 2242793, 2242794, 2242931, 2242974, 2242987, 2244353, 2245247, 2245272, 2245273, 2245274, 2245397, 2245438, 2245439, 2245440, 2245689, 2246820, 2246821, 2246964, 2246965, 2247085, 2247086, 2247087, 2248008, 2248009, 2248210, 2248440, 2248441, 2248453, 2251930, 2252945, 2252953, 2254719, 2257726, 2257734, 2258781, 2258803, 2258811, 2265435, 2265443, 2265575, 2265583, 2268493, 2268507, 2269031, 2269058, 2269589, 2269597, 2269600, 2269619, 2271842, 2273101, 2273128, 2273136, 2273756, 2273764, 2273772, 2274248, 2274256, 2274264, 2274272, 2274914, 2274922, 2274930, 2275864, 2275872, 2276410, 2279061, 2279088, 2279126, 2279460, 2279479, 2279487, 2284545, 2284553, 2284782, 2284790, 2287072, 2294338, 2294346, 2294400, 2295377, 2295385, 2295393, 2297795, 2297906, 2297914, 2297922, 2298279, 2298287, 2298295, 2300451, 2301423, 2301431, 2301458, 2302861, 2302888, 2302896, 2302942, 2302950, 2302977, 2303124, 2303132, 2303140, 2303442, 2303450, 2303469, 2303922, 2305062, 2306166, 2306174, 2306182, 2307170, 2307189, 2307197, 2307553, 2307561, 2307588, 2307634, 2307642, 2307650, 2307669, 2307677, 2307723, 2312050, 2312069, 2312077, 2313596, 2314894, 2314908, 2316544, 2320754, 2320762, 2320770, 2321475, 2321483, 2321491, 2326329, 2326337, 2326345, 2326477, 2326485, 2326493, 2331519, 2331527, 2333554, 2333856, 2333864, 2333872, 2334437, 2334445, 2336316, 2339110, 2339129, 2339587, 2339595, 2340763, 2340771, 2341522, 2341603, 2343606, 2343614, 2345366, 2345374, 2345382, 2345854, 2345862, 2348578, 2350459, 2350467, 2351056, 2351064, 2353377, 2353385, 2354144, 2354152, 2354160, 2354349, 2354357, 2354365, 2354926, 2354934, 2354942, 2355663, 2355671, 2355698, 2356422, 2357453, 2357461, 2357488, 2357887, 2357895, 2357909, 2357917, 2357925, 2361264, 2361272, 2361809, 2361817, 2363232, 2363240, 2363259, 2363518, 2363704, 2363712, 2364506, 2364514, 2365286, 2365294, 2365529, 2365537, 2366347, 2366355, 2366363, 2370921, 2373270, 2373289, 2373297, 2374013, 2374021, 2374048, 2374587, 2374595, 2375842, 2375850, 2375869, 2375877, 2377209, 2378043, 2378051, 2378116, 2378124, 2378620, 2378639, 2378841, 2378868, 2379767, 2379775, 2380196, 2380218, 2380722, 2380730, 2384906, 2384914, 2384922, 2385341, 2385368, 2388766, 2388774, 2388839, 2388847, 2389169, 2389177, 2389185, 2389290, 2389304, 2389312, 2391600, 2397307, 2403250, 2403269, 2403277, 2403366, 2403374, 2403382, 2403412, 2403420, 2403439, 2403447, 2405067, 2406020, 2406039, 2407124, 2408228, 2408236, 2409283, 2409291, 2412829, 2415089, 2415968, 2415976, 2415984, 2416786, 2416794, 2416808, 2417049, 2417057, 2417065, 2417189, 2417197, 2417200, 2417219, 2417227, 2417235, 2418002, 2418010, 2418029, 2419300, 2419319, 2419327, 2419335, 2419343, 2419351, 2421674, 2421682, 2421690, 2421828, 2421836, 2423286, 2424258, 2424266, 2424274, 2425483, 2425491, 2429764, 2429772, 2434121, 2434148, 2434156, 2435462, 2435470, 2437899, 2438275, 2438283, 2438658, 2439328, 2439611, 2441829, 2443635, 2443643, 2443937, 2443945, 2444844, 2444852, 2444933, 2444941, 2446065, 2448599, 2448602, 2448610, 2449390, 2449404, 2449765, 2449935, 2449943, 2455404, 2455412, 2455420, 2455439, 2455447, 2455455, 2456575, 2456583, 2456591, 2456605, 2456613, 2456621, 22303140, 66123203  *Exclusions: gestational diabetes*  ICD-9: 641, 642, 643, 644, 645, 646, 647, 648, 650, 651, 652, 653, 654, 655, 656, 657, 658, 659, 660, 661, 662, 663, 664, 665, 666, 667, 668, 669, 670, 763, V27  ICD-10: O10, O11, O12, O13, O14, O15, O16, O21, O22, O23, O24, O25, O26, O28, O29, O30, O31, O32, O33, O34, O35, O36, O37, O40, O41, O42, O43, O44, O45, O46, O47, O48, O60, O61, O62, O63, O64, O65, O66, O67, O68, O69, O70, O71, O72, O73, O74, O75, O85, O86, O87, O88, O89, O90, O91, O92, O94, O95, O98, O99, Z37 | (3) |
| Hypertension (HTN) | | Looking at persons aged 20 and older:  1 hospitalization with a hypertension diagnostic code   OR  2 physician visits in 2 years with hypertension diagnostic code(s) | ICD-9: 401, 402, 403, 404, 405   ICD-10: I10, I11, I12, I13, I15 | (3) |
| Kidney diseases (KID) | Chronic kidney disease (CKD) | 1 hospitalization with a CKD diagnostic code   OR  2 physician visits in 1 year with CKD diagnostic code(s) | ICD-9: 581, 582, 583, 585, 586, 587, 589  ICD-10: N01, N03, N04, N05, N06, N07, N18, N19, N26, N27 | (3) |
|  | Dialysis | 9 or more physician visits within 90 days with dialysis fee item code(s) | Fee Codes: 00308, 00323, 00350, 00351, 00352, 00355, 00356, 00358, 00359, 00361, 00390, 33708, 33723, 33750, 33751, 33752, 33755, 33756, 33758, 33759, 33761, 33790 | (3) |
|  | Kidney transplant | 1 hospitalization with procedure code(s) for kidney transplant | CCI: 675  CCP: 1PC85 | (3) |
| Liver diseases (LVR) | Cirrhosis | 1 hospitalization with a cirrhosis diagnostic code   OR  2 physician visits with cirrhosis diagnostic code(s) in 1 year | ICD-9 for physician visit: 571  ICD-9 for hospitalization: 456.1, 571.2, 571.5  ICD-10: I85.9, I98.2, K70.3, K71.7, K74.6 | (5) |
|  | Decompensated cirrhosis | 1 hospitalization with a decompensated cirrhosis diagnostic code   OR  2 physician visits with a decompensated cirrhosis diagnostic code(s) in 1 year | ICD-9: 456.0, 456.2, 572.2, 572.3, 572.4, 782.4, 789.5  ICD-10: I85.0, I86.4, I98.20, I98.3, K72.1, K72.9, K76.6, K76.7, R17, R18 | (5) |
|  | Chronic liver disease (CLD) | 1 hospitalization with a CLD diagnostic code   OR  2 physician visits with CLD diagnostic code(s) in 1 year | ICD-9: 070.2, 070.3, 070.41, 070.44, 070.51, 070.54, 070.7, 275.0, 573.3  ICD-10: B16, B17.10, B17.11, B18.0, B18.1, B18.2, B19.10, B19.11, B19.20, B19.21, E83.11, K71.6, K75.9  *Note: We modified the case definition to “2 physician visits/year”, as opposed to “1 physician visit”, to allow for a confirmatory visit. We have also removed diagnostic codes that overlap with those in cirrhosis and decompensated cirrhosis.* | (6) |
| Osteoarthritis (OA) | | 1 hospitalization with an OA diagnostic code   OR  2 physician visits with OA diagnostic code(s) in 1 year | ICD-9: 715  ICD-10: M15, M16, M17, M18, M19 | (3) |

Note: Non-AIDS-defining cancers (cases other than Kaposi sarcoma, non-Hodgkin's lymphoma and cervical cancer) were identified from the BC Cancer Agency Registry, and, as such, are not listed above. For non-AIDS-defining cancers-specific mortality cases, we followed Eyawo et al. and used cancers-specific ICD-10 codes C00-D48, excluding C46, C53, C82 and C83 (7).

**Table B.** Disability weight and literature-derived disease severity distribution associated with selected chronic comorbidities.

| **Chronic Comorbidities** | | **Health state/Sequela** | **Disability Weight (95%CI)** | **Severity Distribution (%)** | **Reference and Notes*** |
| --- | --- | --- | --- | --- | --- |
| Alzheimer’s and non-HIV-related dementia (ALZD) | | Dementia, mild | 0.069 (0.046-0.099) | 25.7 | Disability weight (DW) followed Global Burden of Disease Study (GBD) 2019 (8).  Severity distribution (SvD) was derived from ALZD’s baseline SvD (9), and a 12-month SvD progression, based on the Global Deterioration Scale, in a Canadian population (10). |
|  |  | Dementia, moderate | 0.377 (0.252-0.508) | 45.6 |  |
|  |  | Dementia, severe | 0.449 (0.304-0.595) | 28.7 |  |
| Cardiovascular diseases (CVD) | Ischaemic heart disease (IHD)  Acute myocardial infarction (AMI) | Asymptomatic angina due to IHD | 0 (0-0) | 30.5 | DW followed GBD 2019 (8).  SvD was obtained from a large population survey in the US and Australia (11) |
|  |  | Mild angina due to IHD | 0.033 (0.02-0.052) | 24.0 |  |
|  |  | Moderate angina due to IHD | 0.08 (0.052-0.113) | 12.6 |  |
|  |  | Severe angina due to IHD | 0.167 (0.11-0.24) | 33.0 |  |
|  | Congestive heart failure (CHF) | Asymptomatic heart failure | 0 (0-0) | 18.2 |  |
|  |  | Heart failure, mild | 0.041 (0.026-0.062) | 55.4 |  |
|  |  | Heart failure, moderate | 0.072 (0.047-0.103) | 9.9 |  |
|  |  | Heart failure, severe | 0.179 (0.122-0.251) | 16.5 |  |
|  | Cerebrovascular accident (stroke/CVA)  Transient Ischemic Attack (mini stroke/TIA) | Asymptomatic chronic stroke | 0 (0-0) | 14.5 |  |
|  |  | Chronic stroke severity level 1 | 0.019 (0.01-0.032) | 42.5 |  |
|  |  | Chronic stroke severity level 2 | 0.07 (0.046-0.099) | 22.8 |  |
|  |  | Chronic stroke severity level 3 | 0.316 (0.206-0.437) | 15.9 |  |
|  |  | Chronic stroke severity level 4 | 0.55  (0.377-0.707) | 2.8 |  |
|  |  | Chronic stroke severity level 5 | 0.588 (0.411-0.744) | 1.7 |  |
| Chronic obstructive pulmonary disease (COPD) | | Asymptomatic COPD | 0 (0-0) | 0.0 | DW followed GBD 2019 (8).  SvD was obtained from a phone-based survey in Vancouver, based on lung function criteria (FEV1) developed by the Global Initiative for Chronic Obstructive Lung Disease (12). |
|  |  | COPD and other chronic respiratory problems, mild | 0.019 (0.011-0.033) | 55.9 |  |
|  |  | COPD and other chronic respiratory problems, moderate | 0.225 (0.153-0.31) | 37.8 |  |
|  |  | COPD and other chronic respiratory problems, severe | 0.408 (0.273-0.556) | 6.4 |  |
| Diabetes mellitus (DM) | | Generic uncomplicated disease | 0.049 (0.031-0.072) | 60.1 | DW followed GBD 2019 (8).  SvD was derived from Veteran Health Administration cohort study on diabetic foot (13), and high income North American data on diabetic retinopathy and blindness (14,15). |
|  |  | Distance vision, moderate impairment | 0.031 (0.019-0.049) | 12.9 |  |
|  |  | Distance vision, severe impairment | 0.184 (0.125-0.258) | 1.9 |  |
|  |  | Distance vision blindness | 0.187 (0.124-0.26) | 2.1 |  |
|  |  | Diabetic neuropathy | 0.133 (0.089-0.187) | 15.3 |  |
|  |  | Diabetic neuropathy with diabetic foot | 0.15 (0.103-0.208) | 6.3 |  |
|  |  | Diabetic neuropathy with treated amputation | 0.167 (0.114-0.229) | 1.3 |  |
|  |  | Diabetic neuropathy with untreated amputation | 0.282 (0.198-0.379) | 0.0 |  |
| Hypertension (HTN) | | Stage 1-2 CKD with preserved glomerular filtration rate due to HTN | 0 (0-0) | 41.2 | DW followed GBD 2019 (8).  SvD was derived from US National Health and Nutrition Examination Survey (NHANES) data on CKD and end-stage renal disease focusing on those with hypertension (16), and on anemia among people with CKD (17).  *Disclaimer: The data reported here have been supplied by the United States Renal Data System (USRDS). The interpretation and reporting of these data are the responsibility of the author(s) and in no way should be seen as an official policy or interpretation of the U.S. Government.* |
|  |  | Stage 3 CKD without anemia due to HTN | 0 (0-0) | 42.9 |  |
|  |  | Stage 3 CKD and mild anemia due to HTN | 0.004 (0.001-0.008) | 3.0 |  |
|  |  | Stage 3 CKD and moderate anemia due to HTN | 0.052 (0.034-0.076) | 3.0 |  |
|  |  | Stage 3 CKD and severe anemia due to HTN | 0.149 (0.101-0.209) | 3.0 |  |
|  |  | Stage 4 CKD without anemia due to HTN | 0.104 (0.07-0.147) | 2.5 |  |
|  |  | Stage 4 CKD untreated and mild anemia due to HTN | 0.108 (0.072-0.151) | 0.9 |  |
|  |  | Stage 4 CKD untreated and moderate anemia due to HTN | 0.15 (0.103-0.207) | 0.9 |  |
|  |  | Stage 4 CKD untreated and severe anemia due to HTN | 0.237 (0.165-0.324) | 0.9 |  |
|  |  | Stage 5 CKD untreated without anemia due to HTN | 0.569 (0.389-0.727) | 0.6 |  |
|  |  | Stage 5 CKD untreated and mild anemia due to HTN | 0.57 (0.391-0.727) | 0.2 |  |
|  |  | Stage 5 CKD untreated and moderate anemia due to HTN | 0.591 (0.414-0.743) | 0.2 |  |
|  |  | Stage 5 CKD untreated and severe anemia due to HTN | 0.631 (0.456-0.782) | 0.2 |  |
|  |  | End-stage renal disease after transplant due to HTN | 0.024 (0.014-0.039) | 0.1 |  |
|  |  | End-stage renal disease on dialysis without anemia due to HTN | 0.571 (0.398-0.725) | 0.2 |  |
|  |  | End-stage renal disease on dialysis and mild anemia due to HTN | 0.573 (0.403-0.726) | 0.1 |  |
|  |  | End-stage renal disease on dialysis and moderate anemia due to HTN | 0.593 (0.424-0.742) | 0.1 |  |
|  |  | End-stage renal disease on dialysis and severe anemia due to HTN | 0.633 (0.462-0.781) | 0.1 |  |
| Kidney diseases (KID) | CKD (CKD)   Dialysis  Kidney transplant | Asymptomatic, stage 1-2 CKD with preserved glomerular filtration rate | 0 (0-0) | 45.9 | DW followed GBD 2019 (8).  SvD was derived from US National Health and Nutrition Examination Survey (NHANES) data on CKD and end-stage renal disease (16), and on anemia among people with CKD (17).  *Disclaimer: The data reported here have been supplied by the United States Renal Data System (USRDS). The interpretation and reporting of these data are the responsibility of the author(s) and in no way should be seen as an official policy or interpretation of the U.S. Government.* |
|  |  | Asymptomatic, stage 3 CKD without anemia | 0  (0-0) | 40.8 |  |
|  |  | Stage 3 CKD and mild anemia | 0.004 (0.001-0.008) | 2.9 |  |
|  |  | Stage 3 CKD and moderate anemia | 0.052 (0.034-0.076) | 2.9 |  |
|  |  | Stage 3 CKD and severe anemia | 0.149 (0.101-0.209) | 2.9 |  |
|  |  | Stage 4 CKD untreated without anemia | 0.104 (0.07-0.147) | 1.4 |  |
|  |  | Stage 4 CKD untreated and mild anemia | 0.108 (0.072-0.151) | 0.5 |  |
|  |  | Stage 4 CKD untreated and moderate anemia | 0.15 (0.103-0.207) | 0.5 |  |
|  |  | Stage 4 CKD untreated and severe anemia | 0.237 (0.165-0.324) | 0.5 |  |
|  |  | Stage 5 CKD untreated without anemia | 0.569 (0.389-0.727) | 0.3 |  |
|  |  | Stage 5 CKD untreated and mild anemia | 0.57 (0.391-0.727) | 0.1 |  |
|  |  | Stage 5 CKD untreated and moderate anemia | 0.591 (0.414-0.743) | 0.1 |  |
|  |  | Stage 5 CKD untreated and severe anemia | 0.631 (0.456-0.782) | 0.1 |  |
|  |  | End-stage renal disease after transplant | 0.024 (0.014-0.039) | 0.4 |  |
|  |  | End-stage renal disease on dialysis without anemia | 0.571 (0.398-0.725) | 0.4 |  |
|  |  | End-stage renal disease on dialysis and mild anemia | 0.573 (0.403-0.726) | 0.1 |  |
|  |  | End-stage renal disease on dialysis and moderate anemia | 0.593 (0.424-0.742) | 0.1 |  |
|  |  | End-stage renal disease on dialysis and severe anemia | 0.633 (0.462-0.781) | 0.1 |  |
| Liver diseases (LVR) | Cirrhosis  Decompensated cirrhosis  Chronic liver disease (CLD) | Asymptomatic | 0 (0-0) | 73.5 | DW followed GBD 2019 (8).  SvD was derived using data from an outpatient clinic in Austria on anemia among advanced chronic liver disease (18). The proportion of people with decompensated cirrhosis was obtained from our study data. |
|  |  | Decompensated cirrhosis of the liver | 0.178 (0.123-0.25) | 9.0 |  |
|  |  | Decompensated cirrhosis of the liver and mild anemia | 0.181 (0.126-0.252) | 12.4 |  |
|  |  | Decompensated cirrhosis of the liver and moderate anemia | 0.22 (0.156-0.298) | 3.7 |  |
|  |  | Decompensated cirrhosis of the liver and severe anemia | 0.3 (0.212-0.404) | 1.3 |  |
| Osteoarthritis | | Asymptomatic osteoarthritis | 0 (0-0) | 24.7 | DW followed GBD 2019 (8).  SvD was derived from survey data from British Columbia  (19). |
|  |  | Mild osteoarthritis | 0.023 (0.013-0.037) | 31.1 |  |
|  |  | Moderate osteoarthritis | 0.079 (0.054-0.11) | 30.6 |  |
|  |  | Severe osteoarthritis | 0.165 (0.112-0.232) | 13.5 |  |
| Non-AIDS defining cancer | | Diagnosis and primary therapy phase of cancer | 0.288 (0.193-0.399) | 25.6 | DW followed GBD 2019 (8).  SvD was derived from a Canada-wide report, excluding AIDS-defining cancers (20). |
|  |  | Metastatic phase of cancer | 0.451 (0.307-0.6) | 17.5 |  |
|  |  | Terminal phase of cancer | 0.54 (0.377-0.687) | 19.8 |  |
|  |  | Controlled phase of cancer | 0.049 (0.031-0.072) | 37.2 |  |

Note: *Given administrative data’s inability to ascertain disease severity, we conducted a literature review to obtain comorbidity-specific severity distributions. We searched the MEDLINE database using keywords including “[comorbidity name] severity distribution” OR “[comorbidity name] severity level” OR “[comorbidity name] severity” OR “[comorbidity name] stages”. Simultaneously, internet searches for reports using the abovementioned keywords were conducted. Priority was given to studies from British Columbia (BC), followed by studies from Canada and, subsequently, by studies from settings comparable to BC such as the United States, Australia, or countries in Western Europe. When multiple relevant disease severity distributions were found, priority was given to the distribution whose categories best match the severity categories used in the Global Burden of Disease 2019.

**Table C.** Reference life table or theoretical minimum risk life table (TMRLT) used in GBD 2019 to calculate YLL. The table includes estimates for life expectancy at age x for ages 0 to ≥95 at five-year (21).

| Age (years) | Life Expectancy (years) |
| --- | --- |
| 0 | 88.9 |
| 1-4 | 88.0 |
| 5-9 | 84.0 |
| 10-14 | 79.0 |
| 15-19 | 74.1 |
| 20-24 | 69.1 |
| 25-29 | 64.1 |
| 30-34 | 59.2 |
| 35-39 | 54.3 |
| 40-44 | 49.3 |
| 45-49 | 44.4 |
| 50-54 | 39.6 |
| 55-59 | 34.9 |
| 60-64 | 30.3 |
| 65-69 | 25.7 |
| 70-74 | 21.3 |
| 75-79 | 17.1 |
| 80-84 | 13.2 |
| 85-89 | 10.0 |
| 90-94 | 7.6 |
| ≥95 | 5.9 |

Note: GBD: Global Burden of Diseases Study; YLL: Years of Life Lost due to premature mortality.

**Table D.** Descriptive baseline characteristics of a 1:4 matched analytical sample of PLWH and HIV-negative individuals in British Columba enrolled in the COAST cohort during 2001-2012.

| **Variables** | | **PLWH** | **HIV-negative** |
| --- | --- | --- | --- |
|  |  | **N = 8,031** | **N = 32,124** |
| **Sex at birth, n (%)** | Male | 6612 (82) | 26448 (82) |
|  | Female | 1419 (18) | 5676 (18) |
| **Age at baseline, n (%)** | < 30 | 934 (12) | 3746 (12) |
|  | 30-39 | 2861 (36) | 11397 (35) |
|  | 40-49 | 2786 (35) | 11177 (35) |
|  | ≥ 50 | 1450 (18) | 5804 (18) |
| **Age at baseline (years), median (Q1-Q3)** | | 40 (34-47) | 40 (34-47) |
| **Follow-up time (years), median (Q1-Q3)*** | | 9 (5-12) | 11 (6-12) |
| **History of injection drug use, n (%)** | PWID | 3075 (38) |  |
|  | Non-PWID | 2829 (35) | Not available |
|  | Unknown | 2127 (26) |  |
| **Ethnicity, n (%)** | White | 3028 (38) |  |
|  | Non-White | 1580 (20) | Not available |
|  | Unknown | 3423 (43) |  |

Note: PLWH: people living with HIV; Q1-Q3: 25th-75th percentiles; PWID: PLWH who have ever injected drugs; Non-IDU: PLWH without a history of injection drug use; *: Significant difference in the follow-up time between PLWH and HIV-negative controls (p-value <0.05).

**Table E.** Descriptive baseline characteristics of an analytical sample of PLWH in British Columba, Canada receiving ART through the Drug Treatment Program during 2013-2020.

| **Variables** | | **PLWH** |
| --- | --- | --- |
|  |  | **N = 9,601** |
| **Sex at birth, n (%)** | Male | 7936 (83) |
|  | Female | 1665 (17) |
| **Age at baseline, n (%)** | < 30 | 900 (9) |
|  | 30-39 | 1851 (19) |
|  | 40-49 | 3100 (32) |
|  | ≥ 50 | 3750 (39) |
| **Age at baseline (years), median (Q1-Q3)** | | 47 (38-54) |
| **Follow-up time (years), median (Q1-Q3)** | | 8 (5-8) |
| **History of injection drug use, n (%)** | PWID | 2731 (28) |
|  | Non-PWID | 4354 (45) |
|  | Unknown | 2516 (26) |
| **Ethnicity, n (%)** | White | 4069 (42) |
|  | Non-White | 2038 (21) |
|  | Unknown | 3494 (36) |

Note: PLWH: people living with HIV; Q1-Q3: 25th-75th percentiles; PWID: PLWH who have ever injected drugs; Non-IDU: PLWH without a history of injection drug use; *: Significant difference in the follow-up time between PLWH and HIV-negative controls (p-value <0.05).

**Fig A.** Flowchart outlining derivation of final 1:4 matched analytical sample of 8,031 PLWH and 32,124 HIV-negative individuals in British Columba, Canada (2001-2012).


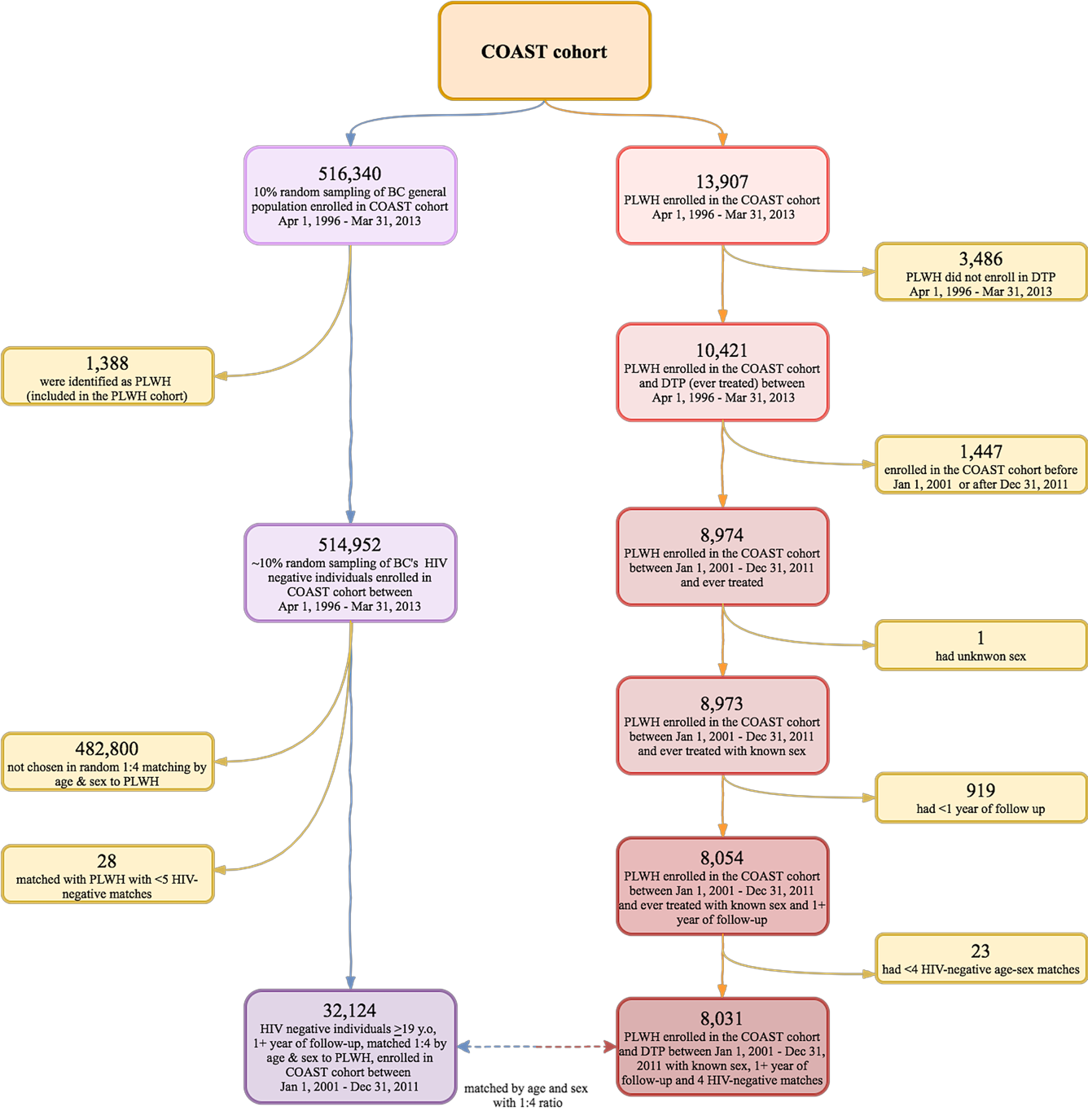


Note: PLWH: people living with HIV; COAST: Comparative Outcomes and Service Utilization Trends; DTP: BC Centre for Excellence in HIV-AIDS Drug Treatment Program; ART: antiretroviral therapy.

**Fig B.** Population pyramids depicting age and sex distribution among ART-treated people living with HIV in British Columbia, Canada for the year 2002 (A), 2012 (B) and 2020 (C).


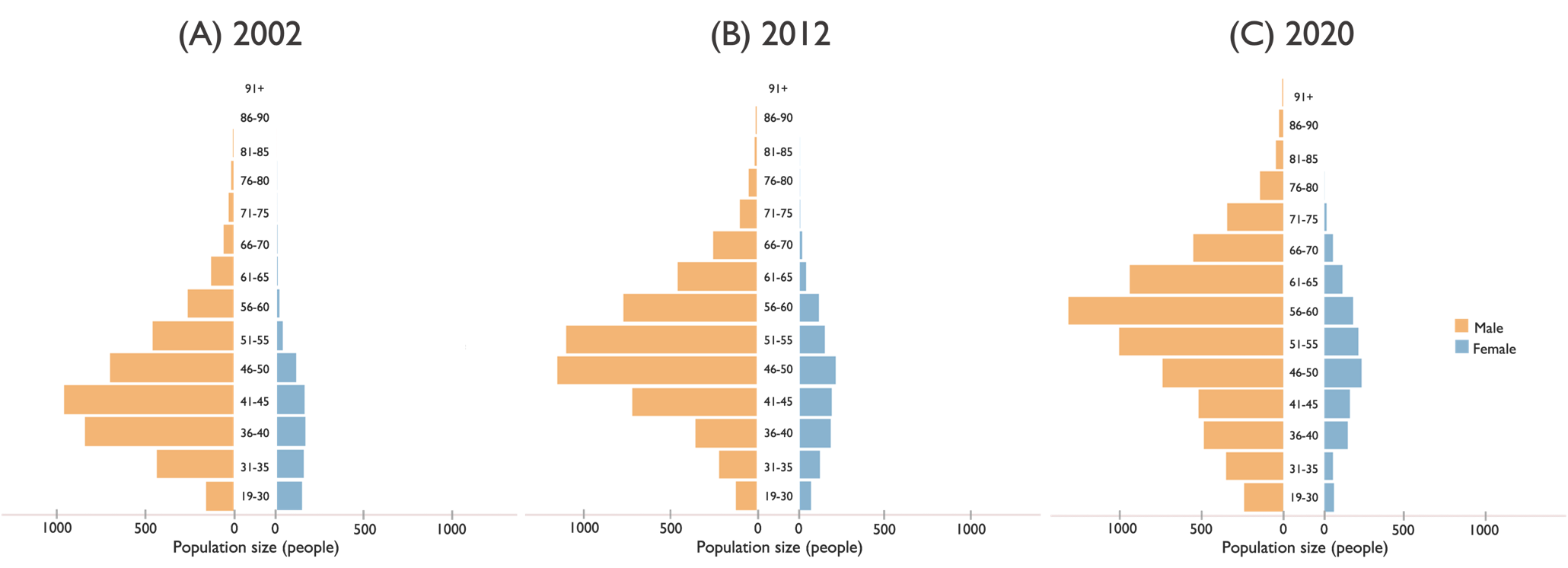


Note: ART: antiretroviral therapy. For 2002 and 2012, data were obtained from the Comparative Outcomes and Service Utilization Trends (COAST) study, while for 2020, data were obtained from the BC Centre for Excellence in HIV-AIDS Drug Treatment Program (DTP) dataset.


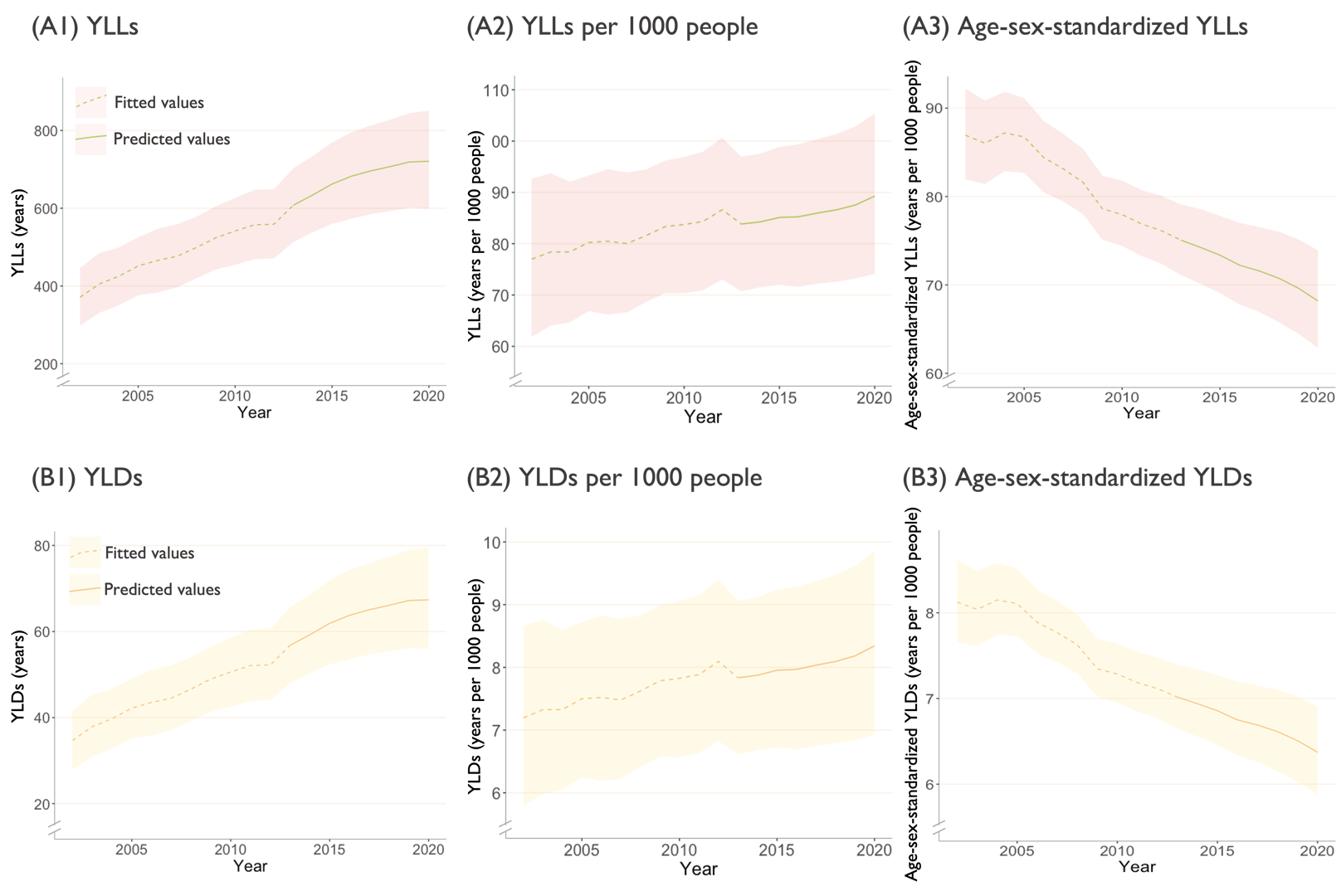
**Fig C.** Fitted (2002-2012) and predicted (2013-2020) YLLs (A) and YLDs (B), with 95% credible intervals, associated with non-AIDS-defining cancers among people living with HIV in British Columbia, Canada.

Note: YLLs: years of life lost due to premature mortality; YLDs: years of healthy life lost due to disability. Vertical scales differ for each graph for illustration purposes.

**References**

1. Cao B, Stevens G, Ho J, et al. WHO methods and data sources for global burden of disease estimates 2000-2019. Geneva: 2020.

2. Hilderink HBM, Plasmans MHD, Snijders BEP, et al. Accounting for multimorbidity can affect the estimation of the Burden of Disease: a comparison of approaches. *Arch. Public Heal.* [electronic article]. 2016;74(1). (/pmc/articles/PMC4993005/). (Accessed January 28, 2022)

3. Chronic Disease Information Working Group. BC Chronic Disease and Selected Procedure Case Definitions version 2016, last updated February 2018. 2015;

4. Chronic Disease Information Working Group. BC Chronic Disease and Selected Procedure Case Definitions version 2017, last updated April 2019. 2015;(http://www.bccdc.ca/health-professionals/data-reports/chronic-disease-dashboard#Case--Definitions)

5. Lapointe-Shaw L, Georgie F, Carlone D, et al. Identifying cirrhosis, decompensated cirrhosis and hepatocellular carcinoma in health administrative data: A validation study. *PLoS One* [electronic article]. 2018;13(8):e0201120. (https://dx.plos.org/10.1371/journal.pone.0201120). (Accessed August 9, 2019)

6. Roberts HW, Utuama OA, Klevens M, et al. The Contribution of Viral Hepatitis to the Burden of Chronic Liver Disease in the United States. *Am. J. Gastroenterol.* [electronic article]. 2014;109(3):387–393. (http://insights.ovid.com/crossref?an=00000434-201403000-00015). (Accessed August 14, 2019)

7. Eyawo O, Franco-Villalobos C, Hull MW, et al. Changes in mortality rates and causes of death in a population-based cohort of persons living with and without HIV from 1996 to 2012. *BMC Infect. Dis.* 2017;17(1):1–15.

8. Global Burden of Disease Collaborative Network. Global Burden of Disease Study 2019 (GBD 2019) Disability Weights. Seattle, United States of America: 2020.

9. Sambrook R, Herrmann N, Hébert R, et al. Special Geriatric Psychiatry Section Canadian Outcomes Study in Dementia: Study Methods and Patient Characteristics. 2004.

10. Herrmann N, Harimoto T, Balshaw R, et al. Risk factors for progression of Alzheimer disease in a Canadian population: The Canadian Outcomes Study in Dementia (COSID). *Can. J. Psychiatry* [electronic article]. 2015;60(4):189–199. (www.TheCJP.ca). (Accessed April 16, 2021)

11. Burstein R, Fleming T, Haagsma J, et al. Estimating distributions of health state severity for the global burden of disease study. *Popul. Health Metr.* [electronic article]. 2015;13(1):31. (http://pophealthmetrics.biomedcentral.com/articles/10.1186/s12963-015-0064-y). (Accessed May 3, 2021)

12. Buist AS, McBurnie MA, Vollmer WM, et al. International variation in the prevalence of COPD (The BOLD Study): a population-based prevalence study. *Lancet*. 2007;370(9589):741–750.

13. Helmer D, Tseng C-L, Wrobel J, et al. Assessing the risk of lower extremity amputations using an administrative data-based foot risk index in elderly patients with diabetes*.

14. Leasher JL, Bourne RRA, Flaxman SR, et al. Global estimates on the number of people blind or visually impaired by diabetic retinopathy: A meta-analysis from 1990 to 2010. *Diabetes Care* [electronic article]. 2016;39(9):1643–1649. (http://care.diabetesjournals.org/lookup/suppl/doi:10.2337/dc15-2171/-/DC1.http://www.anglia.ac.uk/epidemiology /.). (Accessed April 20, 2021)

15. Zhang X, Saaddine JB, Chou CF, et al. Prevalence of diabetic retinopathy in the United States, 2005-2008. *JAMA - J. Am. Med. Assoc.* [electronic article]. 2010;304(6):649–656. (https://jamanetwork.com/). (Accessed April 21, 2021)

16. United States Renal Data System. 2010 USRDS Annual Data Report: Atlas of chronic kidney disease and end-stage renal disease in the United States. Bethesda, MD: 2010.

17. Stauffer ME, Fan T. Prevalence of Anemia in Chronic Kidney Disease in the United States. *PLoS One* [electronic article]. 2014;9(1):e84943. (https://dx.plos.org/10.1371/journal.pone.0084943). (Accessed April 30, 2021)

18. Scheiner B, Semmler G, Maurer F, et al. Prevalence of and risk factors for anaemia in patients with advanced chronic liver disease. *Liver Int.* [electronic article]. 2020;40(1):194. (/pmc/articles/PMC6973120/). (Accessed February 2, 2022)

19. Li L. British Columbia Osteoarthritis Survey. Vancouver, Canada: 2008.

20. Canadian Cancer Statistics Advisory Committee. Canadian Cancer Statistics 2018. Toronto, Canada: 2018.(cancer.ca/Canadian-Cancer-Statistics-2018-EN)

21. Global Burden of Disease Collaborative Network. Global Burden of Disease Study 2019 (GBD 2019) Reference Life Table. Seattle, United States of America: 2020.(http://ghdx.healthdata.org/gbd-2019)
